# Genome-wide survey and characterization of transcription factors in the silk gland of the silkworm, *Bombyx mori*

**DOI:** 10.1371/journal.pone.0259870

**Published:** 2021-11-11

**Authors:** Yan Ma, Qiwei Sun, Lihua Huang, Qin Luo, Wenhui Zeng, Yao Ou, Jingwen Ma, Hanfu Xu

**Affiliations:** 1 State Key Laboratory of Silkworm Genome Biology, College of Sericulture, Textile and Biomass Sciences, Southwest University, Chongqing, China; 2 Department of Paediatrics, Faculty of Medicine, The Chinese University of Hong Kong, Hong Kong, China; 3 Centre for Cardiovascular Genomics and Medicine, Faculty of Medicine, The Chinese University of Hong Kong, Hong Kong, China; USDA Agricultural Research Service, UNITED STATES

## Abstract

Transcription factors (TFs) are key proteins that modulate gene transcription and thereby lead to changes in the gene expression profile and the subsequent alteration of cellular functions. In the silk gland (SG) of silkworm *Bombyx mori*, an important silk-producing insect, TFs are of vital importance in the regulation of silk protein synthesis in this organ. However, which TFs exist and express in the SG remains largely unknown. Here, we report the large-scale identification of TFs in the SG based on available full-length transcript sequences and the most recent version of silkworm genome data. In total, 348 candidate TFs were identified by strict filtration and were classified into 56 TF families. Chromosomal distribution, motif composition, and phylogenetic relationship analyses revealed the typical characteristics of these TFs. In addition, the expression patterns of 348 TFs in various tissues of *B*. *mori*, especially the SG of fourth-molt (4LM) and day-3 and day-4 fifth-instar (5L3D and 5L4D) larvae, were investigated based on public RNA-seq data and gene microarray data, followed by spatiotemporal verification of TF expression levels by quantitative real-time PCR (qRT-PCR). This report describes the first comprehensive analysis of TFs in the *B*. *mori* SG. The results can serve as a baseline for further studies of the roles of TFs in the *B*. *mori* SG.

## Introduction

Transcription factors (TFs) are master regulators that recognize and bind to specific DNA sequences that are usually located in the 5’-upstream regions of target genes to modulate gene expression; they thereby exert control over processes that specify cell types and developmental patterning and control specific signaling pathways [1–3]. The ability of TFs to regulate gene expression usually depends solely on their ability to bind to specific DNA sequences. Dysfunctions in TFs or mutations in TF-binding sites underlie a series of developmental disorders and diseases [3]. TFs can function individually, but many of them show a tendency to physically interact with other TFs and/or cofactors to form homo- or heterodimers and even larger complexes, resulting in the activation or inhibition of gene transcription and allowing subsequent tissue- and developmental stage-specific gene expression [4, 5]. In view of the crucial, diverse roles of TFs in regulating the growth and development of organisms, continued efforts to identify TFs as well as the DNA sites to which they preferentially bind and to reveal the details of their roles and interactions are necessary to better understand TF-mediated gene regulatory mechanisms.

The silk gland (SG) is a unique specialized organ that synthesizes two major components of silk proteins: the aqueous protein sericin, synthesized in the middle region of the gland (middle silk gland, MSG), and the fibrous protein fibroin, synthesized in the posterior region (posterior silk gland, PSG) [6]. The SG first appears in the early embryonic stage, grows slowly with body growth during the first- to fourth larval instars, and then grows rapidly in the fifth larval instar [7, 8]. Silk protein synthesis in the SG is turned on by regulatory factors during feeding stages, especially in the last larval stage, but is repressed in all molting stages [9, 10]. It is of great significance to explore how silk protein synthesis is spatiotemporally regulated during SG development, which could accelerate the genetic modification of silk properties and contribute to the study of organ development regulation.

In *B*. *mori*, emerging evidence has revealed that TFs are indispensable for the regulation of SG development and silk protein synthesis. For example, Sex combs reduced (Scr) and Antennapedia (Antp) have been proposed to regulate SG organogenesis [11, 12]. Silk gland factor 1 (SGF1), SGF3, and Antp have been shown to regulate sericin protein synthesis [13–15]. Fibroin-modulator-binding protein 1 (FMBP1), SGF2, Dimm, Sage, POU-M2 and βFTZ-f1 have been found to regulate fibroin protein synthesis [16–21]. Although much effort has been put forth in this context, the overall picture of the involvement TFs in the regulation of SG development and silk protein synthesis is far from clear.

In this study, we report the rigorous in-depth identification of TFs from *B*. *mori* SG based on available sequencing data, including assembled full-length transcriptome data, RNA-seq data, gene microarray data, and the most recent complete silkworm genome sequence. We analyzed the characteristics of the TFs in the SG, including their subgroup classifications, chromosomal locations, motif compositions, evolutionary relationships, and expression profiles. This work provides valuable information for better understanding the overall role of TFs in the SG and for further research concerning the roles of TFs in regulating SG development and silk protein synthesis.

## Materials and methods

### Identification of putative TFs in the SG

**Fig 1:**
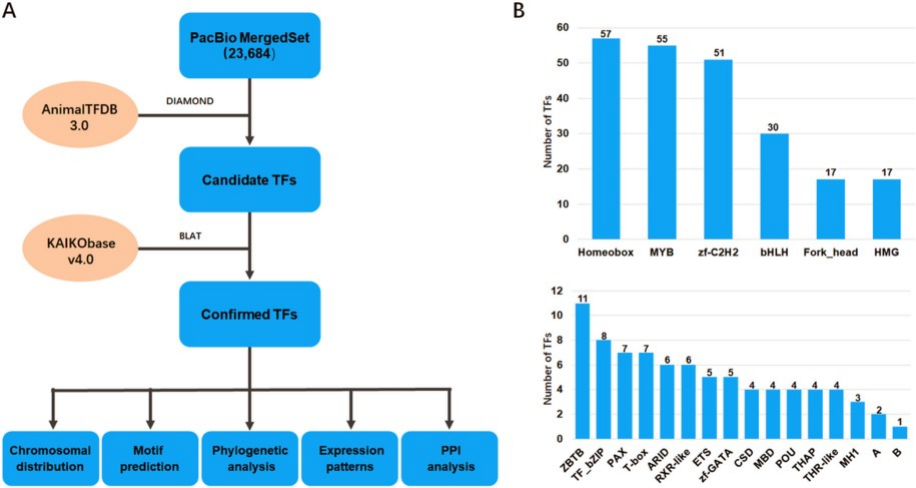
Distribution of TF families identified in the SG. (A) The pipeline of TF identification and analysis. (B) DBD families of TFs in the SG. The x-axis presents the TF family type, and the y-axis presents the number of TFs. The capital letter “A” indicates that the DBD families contain only two TFs, including CP2, DM, E2F, RHD, SF-like, TSC22, and zf-LITAF-like. “B” represents DBD families with only one TF, including AF-4, CBF, COE, CSL, CUT, DACH, ESR-like, GCM, GCNF-like, HPD, HSF, HTH, Miscellaneous, NDT80/PhoG, NF-YB, NGFIB-like, Nrf1, PC4, RFX, Runt, SAND, SRF, TEA, Tub, zf-BED, zf-C2HC, zf-CCCH, and zf-MIZ, etc.

The pipeline of TF prediction and analysis is shown in Fig 1A. First, amino acid sequences deduced from the assembled full-length SG transcript sequences, which we obtained previously from mixtures of five SG samples collected from day-3 fourth-instar larvae and day-1, day-3, day-5, and day-6 fifth-instar larvae [22], were aligned against the AnimalTFDB database (v3.0, http://bioinfo.life.hust.edu.cn/AnimalTFDB/#!/) by using the DIAMOND program (v2.0.4.142, [23]) with strict filters: percentage of identical matches > = 50 and e-value< = 10–5. Then, the IDs of candidate TFs were mapped to the most recent complete silkworm genome deposited in KAIKObase (v4.0, https://kaikobase.dna.affrc.go.jp/) based on the correlations between the old IDs (with prefix of ‘BMgn’) and the new IDs (with prefix of ‘KWMTBOMO’) provided in KAIKObase; otherwise, BLAT (v.36×2; [24]) was applied according to the criterion of a 75% match percentage. A manually modified GFF file (S1 File) based on the KAIKObase v4.0 genome was thus generated for RNA-seq expression quantification.

#### Chromosomal distribution and motif prediction

The chromosomal distribution map of TFs was generated based on the most recent size information for *B*. *mori* chromosomes using online MapChart 2.32 software [25, 26]. Putative conserved motifs of TFs were predicted with the online MEME program [27] (https://meme-suite.org/meme/tools/meme) using the following parameters: maximum number of motifs, 10; minimum motif width, 5; and maximum motif width, 50. Then, the motifs were manually filtered with e-value< = 10^−5^.

### Phylogenetic analysis

Alignment of the amino acid sequences of *B*. *mori* TFs and corresponding TFs in *Drosophila melanogaster* were conducted using multiple protein sequence alignment (MUSCLE, http://www.drive5.com/muscle/) [28]. Phylogenetic trees were constructed by the neighbor-joining (NJ) method with 1,000 bootstrap replicates using MEGA X software, and evolutionary distances were computed by using the p-distance method [29–31]. All ambiguous positions in each sequence pair were filtered out (i.e. pairwise deletion option). The color of each branch was added by iTOL online software [32], the genes in sub branch element with a high boost values give a high confidence to be paralog.

### Microarray data analysis

Gene microarray data of different tissues from day-3 fifth-instar larvae of *B*. *mori* [33] were used to investigate the tissue expression profiles of TFs. These tissues included testis (TE), ovary (OV), head (HD), integument (IG), fat body (FB), midgut (MG), hemocyte (HE), Malpighian tubule (MT), A/MSG (a mixture of anterior silk gland (ASG) and MSG), and PSG. A gene was considered expressed at a point if the signal intensity was >400. The expression array figures were constructed by applying the Cluster 3.0 tool and visualized in TreeView.

### RNA-seq data analysis

Public raw RNA-seq data with accession numbers SRP091606, SRP131538 and SRP072722 [34–36] were downloaded from the NCBI Short Read Archive (SRA, http://www.ncbi.nlm.nih.gov/sra/) and used for RNA-seq meta-analysis. The samples that were sequenced to generate these raw data were of ASG, MSG and PSG collected from fourth-molt (4LM), day-3 fifth-instar (5L3D) and day-4 fifth-instar (5L4D) larvae. All MSG samples were combined for SRP091606. Only wild-type samples were retained for further analysis. All three datasets were aligned against the silkworm genome sequence of KAIKObase v4.0 with subread v1.6.5 [37] and parameters -t 0 -T 4, followed by the quantification of gene expression using Feature Counts v1.6.5 [38]. Expression profiles of TFs were integrated with ComBat-Seq from the R package sva (v3.38.0, https://doi.org/10.1093/nargab/lqaa078) to remove batch bias. Expressed TFs were filtered according to the requirement of at least one sample with no less than 5 supported reads and normalized by log-counts-per-million with edgeR v3.32.0 [39], and the results were illustrated with the R package pheatmap (v 1.0.12.).

### Quantitative real-time PCR (qRT-PCR)

*B*. *mori* larvae (*Nistari* strain) at the 4LM, 5L3D and 5L4D stages were used to collect tissues. Total RNA of these tissues was isolated using the E.Z.N.A. MicroElute Total RNA Kit (Omega Bio-tek, USA). cDNA templates were obtained by reverse transcription of 2 μg of total RNA using the PrimeScript™ RT Reagent Kit with gDNA Eraser (Takara, Japan). The mRNA levels of candidate TFs were analyzed by qRT-PCR using gene-specific primers (S1 Table) on StepOnePlus™ real-time PCR System (Thermo Fisher, USA). Amplification (reaction mixture: 2 μL of cDNA template, 0.8 μL of gene primers, 10 μL of LSYBR® Premix Ex Taq^TM^, 0.4 of μL ROX Reference Dye, and 6 μL of ddH_2_O) was carried out with a 30 s denaturing cycle at 95°C followed by 40 cycles of 3 s at 95°C, 30 s at 60°C, then 15 s at 95°C, 1 min at 60°C, and 95°C for 15 s. The *B*. *mori* eukaryotic translation initiation factor 4A (*BmeIF4A*; [40]) was used as an internal control. All experiments were carried out with three biological replicates. All analyses were completed using GraphPad Prism 6.0.

## Results

### Summary of TFs identified in the SG

**Fig 2:**
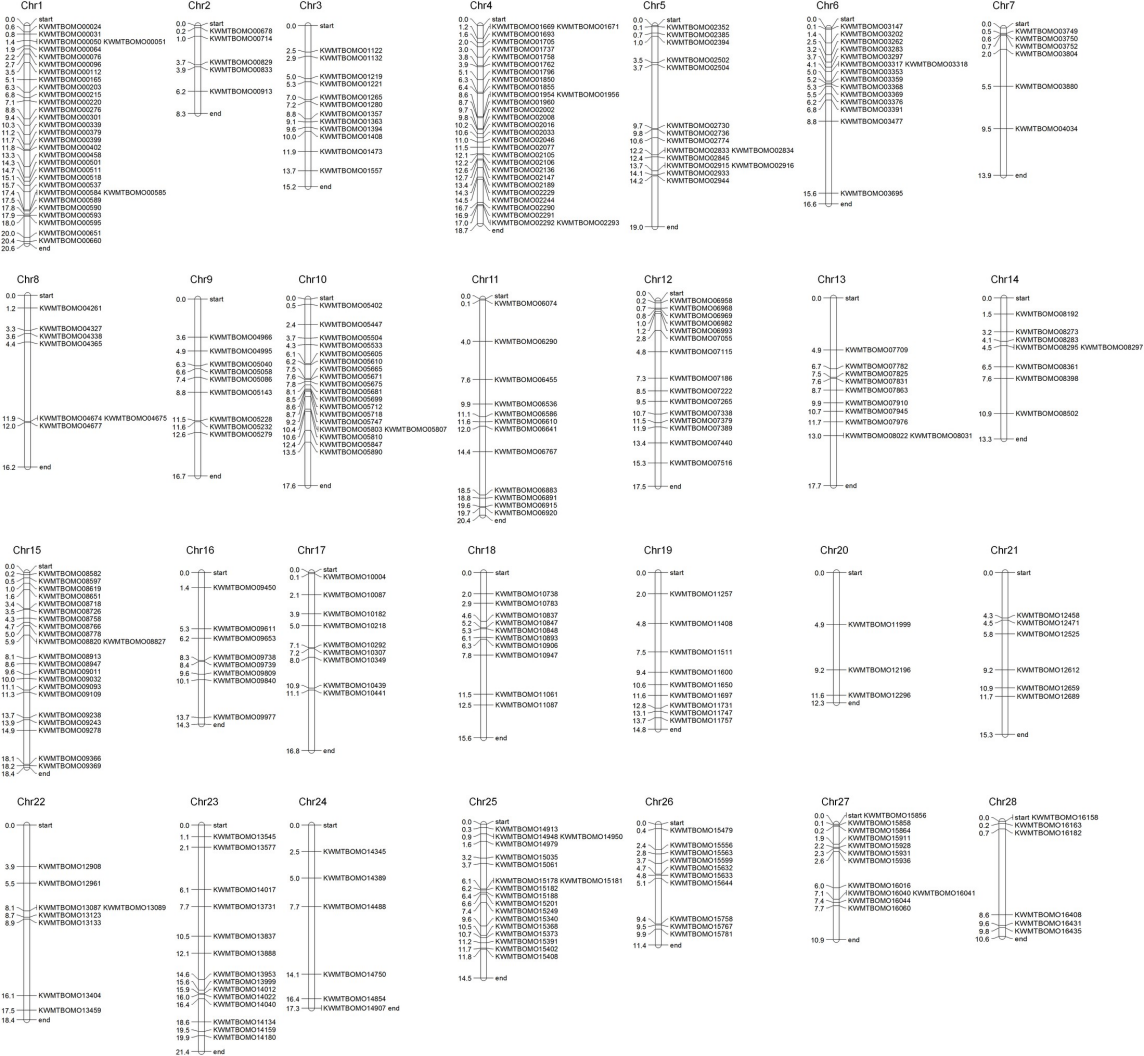
Chromosomal distribution of TFs identified in SG. The distribution map of TFs on 28 chromosomes of *B*. *mori* was constructed using online MapChart 2.32. The abbreviation Chr represents a chromosome. The distance unit on chromosomes is mega bases.

To identify the TFs in the SG of *B*. *mori*, we analyzed the assembled full-length transcript sequences derived from the SG representing five larval development stages combined with the recently released complete genome sequence of *B*. *mori*. As a result, a total of 348 sequences were identified as potential TFs under strict filtration conditions, as summarized in S2 Table. Based on the sequence homology of the DNA-binding domain (DBD), the TFs were further classified into 56 DBD families. The top 6 families were the homeobox (57), MYB (55), zf-C2H2 (51), bHLH (30), Fork_head (17) and HMG (17) families, which accounted for 65.2% of the total TFs (Fig 1B). In addition, 340 of the 348 TFs were shown to be widely and unevenly distributed among 28 chromosomes of *B*. *mori* (Fig 2).

### Motif composition of TFs

**Fig 3:**
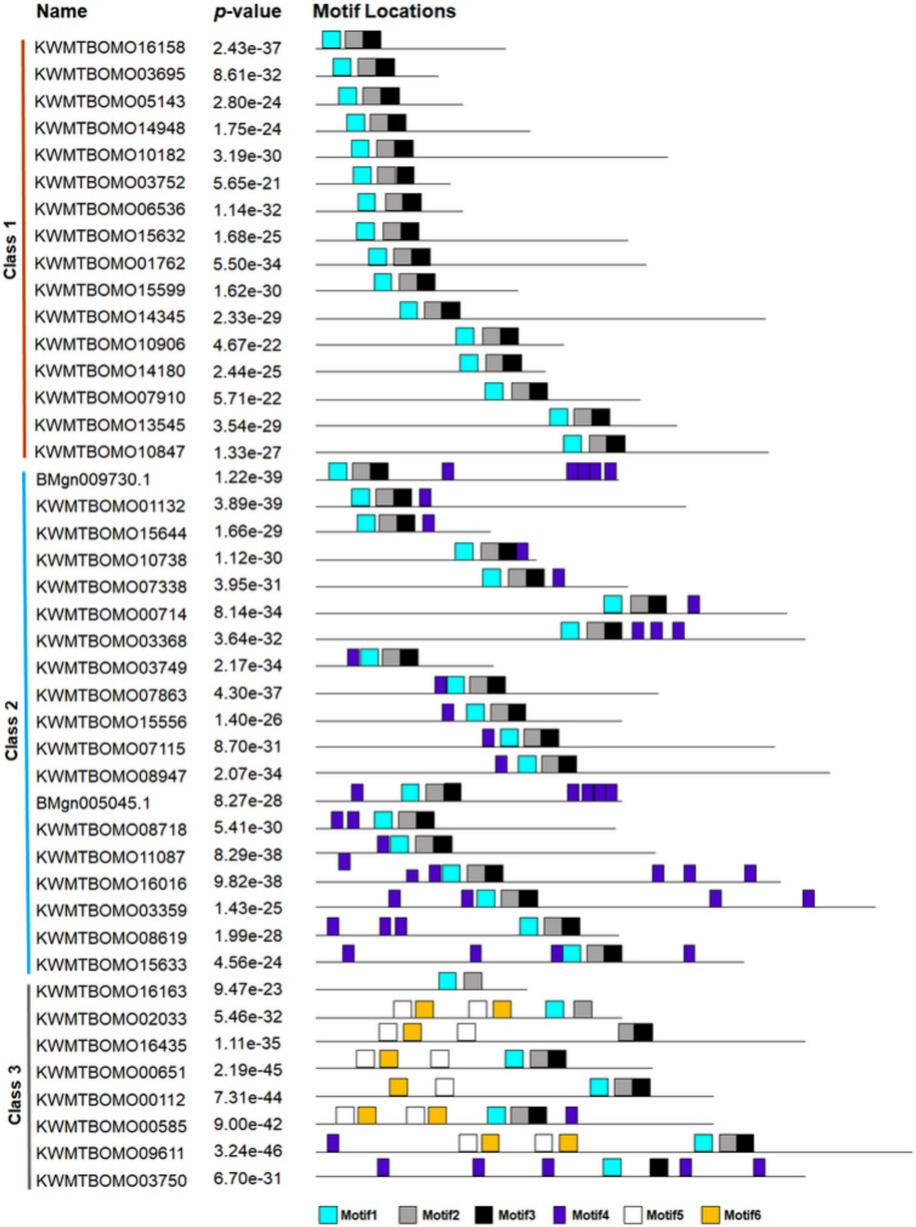
Distribution of conserved motifs of the homeodomain TF family. A total of six conserved motifs were identified in the TFs of the homeodomain family by the online MEME program. The motifs are shown in different colors and numbered 1 to 6. This family was classified into three subgroups based on the motif composition.

Given the regulatory roles of TFs in gene expression associated with specific DBDs (motifs), we used the MEME database to identify the conserved TF motifs of all TF families. The results revealed that putative motifs with E values less than 10^−5^ could be identified for most of the TF families, as summarized in S2 File. Taking the homeodomain TF family as an example (Fig 3), this family was classified into three subgroups based on the motif composition. The highly conserved motifs were Motif 1, Motif 2 and Motif 3, which were distributed across nearly all of the subgroups. Motif 4 was exclusively present in Class 3, while Motif 5 and Motif 6 were mainly shared within Class 2 and Class 3. Interestingly, all members of Class 1 and Class 2 were characterized by a combination of Motif 1, Motif 2 and Motif 3, suggesting that these motifs exhibiting an orientation preference may be related to the specific function of the TFs and are deserving of further study.

### Phylogenetic relationships of TFs

**Fig 4:**
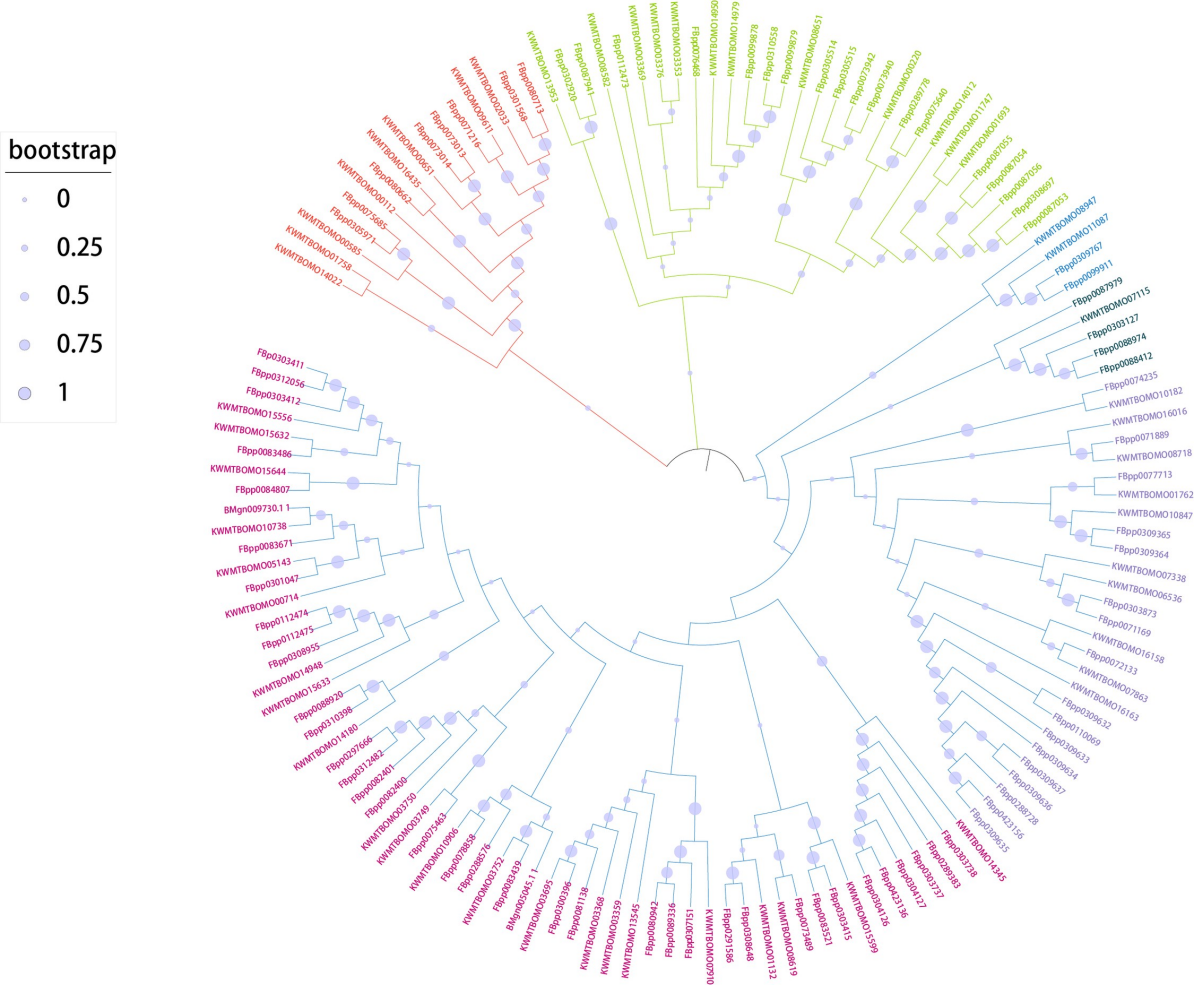
Phylogenetic analysis of the homeodomain TF family. The homeodomain TFs of *B*. *mori* and corresponding proteins of *D*. *melanogaster* were pooled, and alignments of complete TF amino acid sequences were generated using multiple protein sequence alignment (MUSCLE, http://www.drive5.com/muscle/). The phylogenetic tree was constructed via the neighbor-joining method with 1000 bootstrap replicates by using MEGA X. The percentage of replicate trees in which the associated taxa clustered together in the bootstrap test are shown next to the branches. The evolutionary distances were computed using the p-distance method.

To explore the evolutionary and phylogenetic relationships of the TFs, a phylogenetic tree of the members of each of the top 10 TF families was constructed. As revealed in S3 File, although each transcript was annotated to the same protein domain, some branches remained in the middle, and most of the corresponding bootstrap scores were very low, suggesting that the TF similarity of these annotated protein domains with the same conserved regions was high and that the differences among them were not obvious. Here, we take the homeodomain TF family as an example (Fig 4). According to functional category annotation in KAIKObase, the main molecular function of the TFs of the homeodomain family is DNA binding. It can be roughly inferred from the evolutionary diagram that higher bootstrap scores indicate higher similarity in terms of structure and function, probably due to the paralogs resulting from gene duplication. Furthermore, the orthologs between *B*. *mori* and *D*. *melanogaster* are reflected in the tree. For example, KWMTBOMO11747 (Achintya) and KWMTBOMO01693 (LOC100862767 isoform X1) have similar functions, while KWMTBOMO09611 (hypothetical protein B5X24_HaOG207507) and KWMTBOMO02033 (LIM/homeobox protein Lhx3 isoform X2) may have more similar structures and functions. These genes derive from the common ancestor of FBpp0071216 (Lim1), FBpp0080713 and FBpp0301568 (Lim3) in *D*. *melanogaster*.

### Expression pattern analysis of TFs

**Fig 5:**
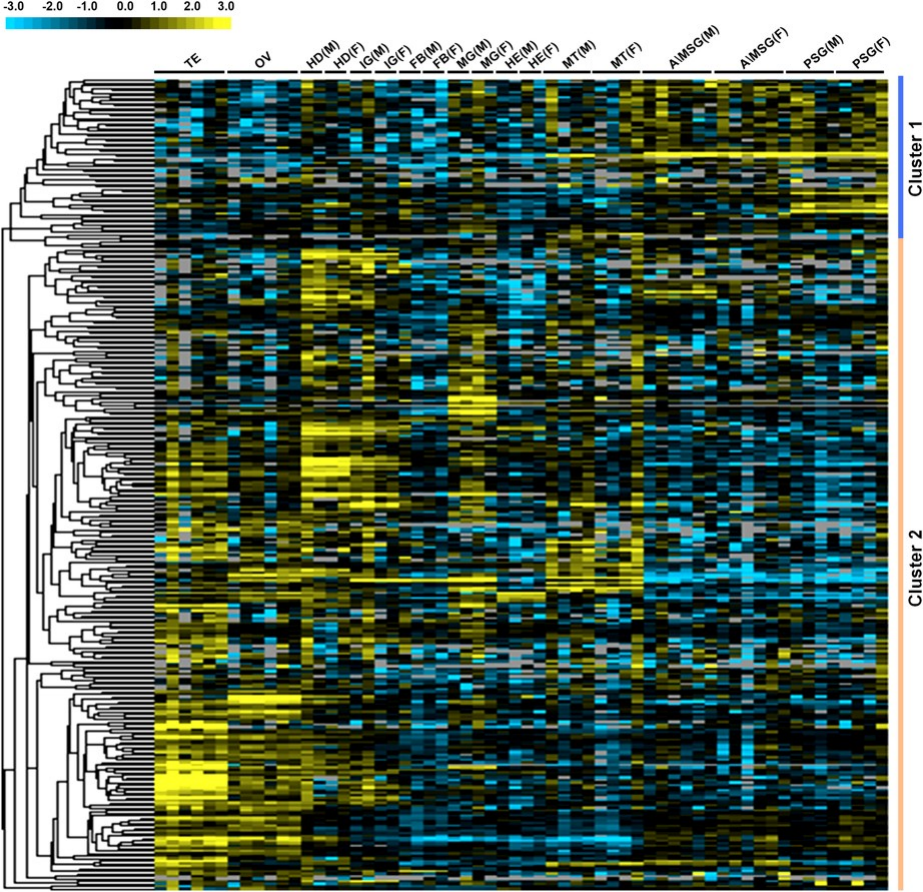
Microarray expression profiling of TFs in multiple tissues of the silkworm. Tissue expression microarray data were obtained from 5L3D silkworm larvae. The expression array figures were constructed by applying the Cluster 3.0 tool and visualized in TreeView. Clusters 1 and 2 are indicated by different colors. The tissues include testis (TE), ovary (OV), head (HD), integument (IG), fat body (FB), midgut (MG), hemocyte (HE), Malpighian tubule (MT), A/MSG (a mixture of ASG and MSG), and PSG. M, male; F, female.

To investigate the expression of the TFs, first, we investigated their tissue expression by analyzing the microarray data sets of *B*. *mori* larvae (5L3D) tissues. As shown in Fig 5, 323 probes out of 348 TFs were detected in the microarray data. Interestingly, the TFs in Cluster 1 were highly expressed in SG, e.g., Dimm, Sage, and SGF1, which have been demonstrated to be key TFs in the regulation of silk protein synthesis [18, 19]. Many TFs in Cluster 2 appeared to be highly active in other tissues, such as the gonad and head, suggesting that these TFs assume regulatory functions in other tissues in 5L3D larvae.

**Fig 6:**
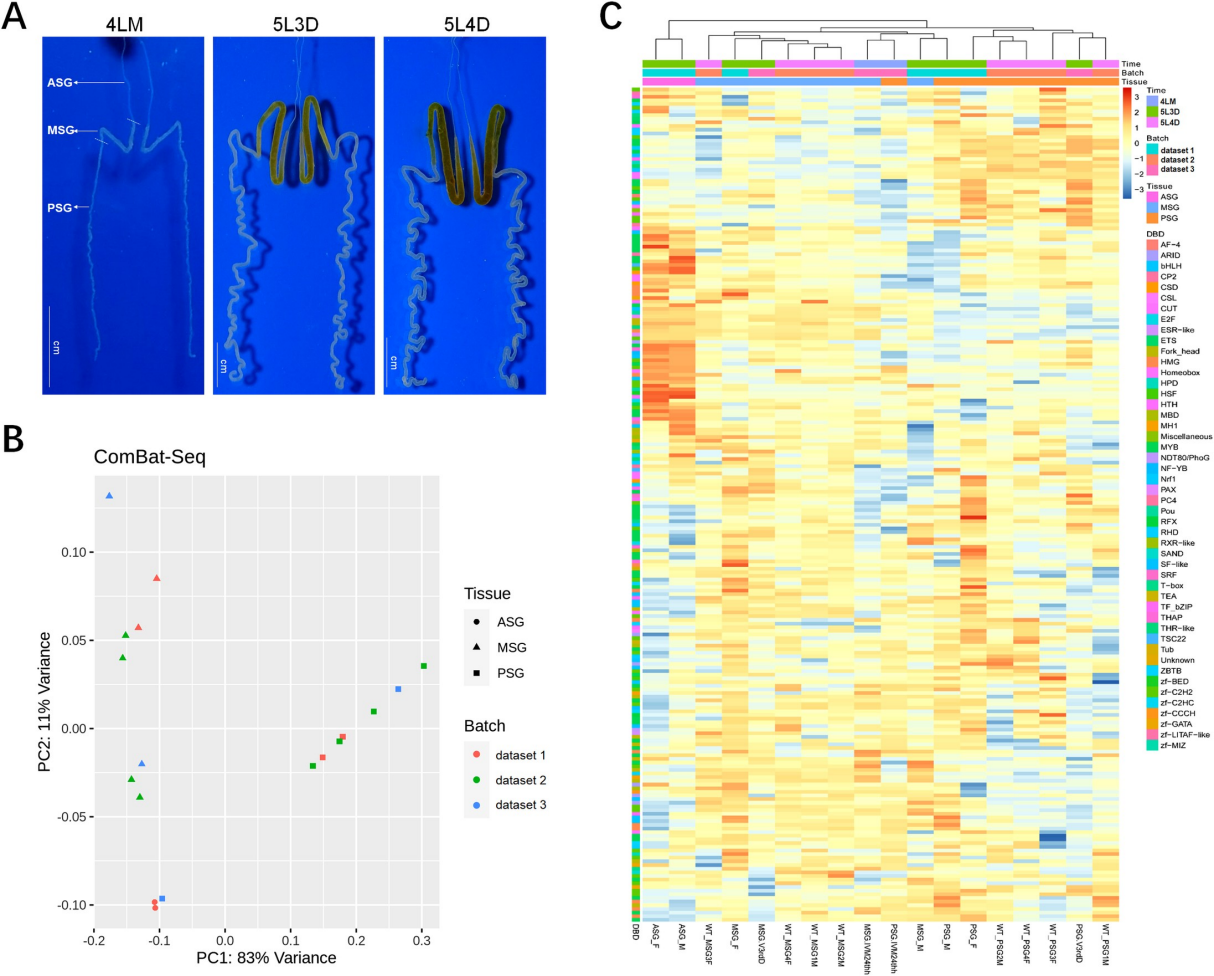
Expression profiles of TFs in the SG representing three larval development stages. (A) SGs dissected from silkworm larvae (strain Nistari) developed to the fourth-molt (4LM), day-3 fifth-instar (5L3D) and day-4 fifth-instar (5L4D) stages. (B) PCA of three published RNAseq datasets, normalized by row and column. (C) Heatmap of 228 TFs expressed in the ASG, MSG, and PSG.

Next, we focused on TF expression in the SG of larvae of different stages. By analyzing the RNA-seq data of ASG, MSG and PSG samples from 4LM, 5L3D and 5L4D larvae (Fig 6A), we found that 228 of 348 TFs were considered expressed in SG (S3 Table). Many of the TFs that were expressed at high levels existed in all three sections of the SG and did not appear to be stage specific (Fig 6B and 6C). In addition, some TFs that exhibited significant differences in expression level between MSG and PSG or between the molting and larval stages were detected, including several TFs that have previously been proven essential for regulating silk protein synthesis, such as SGF1, SGF3 and Awh (Table 1). Interestingly, several members of the Hippo and 20E pathways including Sd, E74B, and HR39, were shown to be highly expressed in both the MSG and PSG of 4LM larvae, while EcR, USP, HR3 and E75A in the 20E pathway appeared to be highly expressed in the PSG of 4LM larvae. These clues strongly suggest that Hippo and 20E signaling plays an important role in the regulation of SG development and silk protein synthesis.

**Table 1:** Expression data of 15 representative TFs expressed in the SG.

Gene ID in silkbase	Name	logCPM (raw read count)							
		MSG_4LM^a^	MSG_5L3D^a^	MSG_5L3D^b^	MSG_5L3D^b^	PSG_4LM^a^	PSG_5L3D^a^	PSG_5L3D^b^	PSG_5L3D^b^
KWMTBOMO15340	Sage	5.59(230)	6.59(616)	6.76(30893)	7.40(24397)	6.19(828)	7.55(1062)	7.10(11053)	7.00(7391)
KWMTBOMO08651	Exd	6.36(392)	6.91(772)	6.96(35304)	5.77(7846)	6.18(818)	6.69(584)	6.89(9547)	6.26(4408)
KWMTBOMO10307	Dimm	4.49(107)	6.74(685)	6.52(26072)	5.55(6765)	3.61(138)	8.25(1718)	8.25(24405)	6.70(5985)
KWMTBOMO05671	E75A	5.46(210)	5.46(282)	5.96(17687)	5.44(6250)	5.60(547)	5.89(335)	6.18(5820)	5.76(3106)
KWMTBOMO01855	USP	5.40(201)	5.95(396)	6.23(21361)	4.38(2994)	5.09(386)	4.56(133)	5.15(2845)	5.46(2534)
KWMTBOMO14979	Hth	4.05(79)	5.56(302)	5.97(17810)	4.78(3958)	5.45(494)	4.83(161)	6.09(5484)	5.41(2447)
KWMTBOMO15391	SGF1	3.42(51)	5.41(272)	5.62(13999)	4.97(4508)	5.03(369)	2.56(33)	5.86(4659)	5.03(1881)
KWMTBOMO13459	SGF3	5.96(296)	4.51(146)	4.74(7584)	4.12(2502)	4.99(360)	-0.80(3)	0.57(116)	-0.37(42)
KWMTBOMO08597	E74B	5.06(159)	3.79(88)	4.51(6444)	3.00(1144)	4.65(283)	3.75(76)	5.27(3101)	4.22(1068)
KWMTBOMO15563	E93	-4.46(0)	4.40(135)	3.84(4070)	2.52(821)	0.99(22)	4.21(104)	4.30(1580)	3.85(827)
KWMTBOMO08031	Sd	3.51(54)	2.33(32)	3.70(3675)	2.78(982)	3.61(138)	2.47(31)	4.23(1500)	3.24(540)
KWMTBOMO00651	Awh	-4.46(0)	-2.31(1)	0.18(308)	-0.33(108)	2.56(66)	4.56(133)	4.39(1685)	4.07(961)
KWMTBOMO05605	EcR	2.61(29)	1.92(24)	3.08(2389)	0.25(165)	2.24(53)	0.15(6)	2.27(385)	0.90(105)
KWMTBOMO08726	HR39	2.34(24)	-0.02(6)	2.25(1341)	1.94(546)	2.04(46)	-0.10(5)	1.49(222)	2.04(234)
KWMTBOMO00829	HR3	0.78(8)	1.05(13)	0.32(343)	-1.09(61)	1.23(26)	-4.46(0)	-1.21(31)	-2.92(5)

^**a**^The raw RNA-seq data in brackets were derived from the SG by Hu *et al*. in 2016.

^**b**^The raw RNA-seq data in brackets were derived from the SG by Chang *et al*. in 2015.

### Expression verification of representative TFs

**Fig 7:**
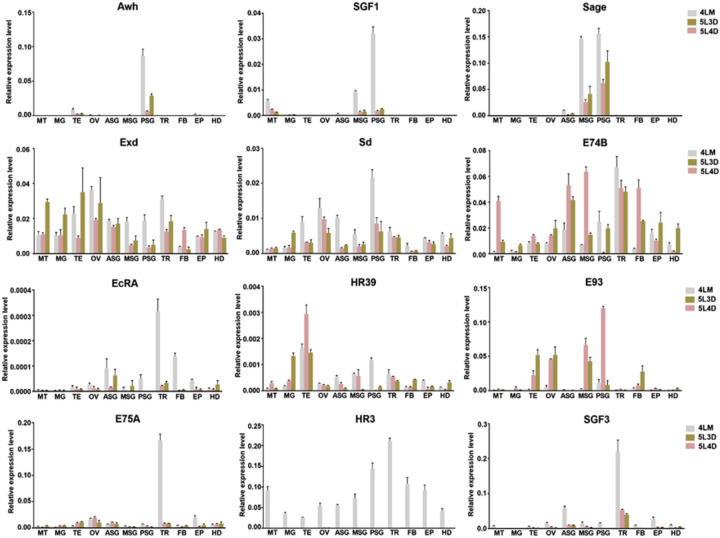
Expression of 12 TFs in different tissues and developmental stages. The expression levels of 12 TFs in different tissues and developmental stages of *B*. *mori* larvae were quantified by qRT-PCR. Relative mRNA levels are calculated as the ratio of target TF mRNA level to BmeIF4A mRNA level. Error bars represent the mean ±SD of three samples. MT, Malpighian tubule; MG, midgut; TE, testis; OV, ovary; ASG, anteriorsilkgland; MSG, middle silk gland; PSG, posterior silk gland; TR, trachea; FB, fat body; EP, epidermis; HD, head. 4LM, the fourth molt; 5L3D, day-3 fifth instar; 5L4D, day-4 fifth instar.

Furthermore, we selected 12 representative TFs, listed in Table 1, and validated their tissue and stage expression in larvae at 4LM, 5L3D and 5L4D by qRT-PCR. As shown in Fig 7, the expression patterns of these TFs could be roughly divided into three types. The first type was TFs that were highly expressed in SG, such as Awh, SGF1 and Sage. The second was TFs that were expressed in most tissues and developmental stages, such as Exd, Sd and E74B. The third was TFs that were highly expressed in tissues of 4LM larvae, such as EcRA, HR3 and SGF3. These results were consistent with the tissue microarray data and RNA-seq data. Notably, the expression patterns of several TFs, including HR3, E93 and SGF3, were very distinct and warrant further study.

## Discussion

Regarding the silk-producing insect *B*. *mori*, the most important and interesting question that has not yet been answered is how it efficiently synthesizes silk proteins in the SG. Increasing evidence shows that TFs, including but not limited to the Dimm, Sage, Awh, FMBP1, SGF1, SGF2, POU-M2 and βFTZ-f1 [16–21], are of vital importance in the regulation of silk protein synthesis. However, it remains largely unclear which TFs function in the SG. Here, we report the identification of 348 TFs in the SG achieved by analyzing the assembled full-length SG transcript sequences that we previously obtained [22]. These TFs were divided into 56 DBD families, among which the top 10 families accounted for 74.7% of all TFs. This work provides useful information for understanding the roles of TFs in the regulation of silk protein synthesis in the SG. It is undeniable that a small number of TFs were missed in this identification because the current full-length transcriptome data do not cover all developmental stages of the SG, which is an issue that should be addressed in the future.

It is meaningful to investigate TF expression in *B*. *mori* tissues, especially the SG, as the expression profiles of TFs can be indicative of their functions. In this study, the tissue microarray analysis revealed that the majority of TFs exhibit non-tissue-specific expression in *B*. *mori* larvae of 5L3D. The expression patterns of the TFs could be divided into two clusters—TFs in Cluster 1 were expressed at high levels in the SG, while TFs in Cluster 2 appeared to be highly expressed in other tissues. The qRT-PCR verification revealed that most of the detected TFs were expressed not only in SG but also in various other tissues. Although the current microarray data only reflect the tissue expression patterns of TFs at one larval stage, these results partially reflect the ubiquitous role of TFs in gene regulation in *B*. *mori* tissues. Extensive experimental evidence shows that some TFs, such as Antp, Scr, and βFTZ-f1, play important roles not only in the SG but also in other tissues and in various biological processes [11, 12, 21]. Nevertheless, detailed investigations are still needed to determine the expression patterns of these TFs in various tissues to clarify their diverse regulatory roles.

To explore the expression patterns of TFs in the SG at different developmental stages, we further analyzed the public RNA-seq data of three kinds of SG samples (ASG, MSG and PSG) collected from *B*. *mori* larvae at the 4LM, 5L3D and 5L4D stages. Interestingly, 228 of 348 TFs were considered expressed in the SG, and many were detected in all three kinds of SG samples and did not appear to be stage specific. Although the RNA-seq data we used here covers only three developmental stages of the SG, it is not surprising that the expression of many of the TFs can be detected in the SG at different stages. As revealed by qRT-PCR detection, 11 of 12 TFs were expressed in the SG of 4LM, 5L3D and 5L4D larvae; only HR3 was expressed mainly in SG at the molting stage. Overall, these results and insights represent the basic but crucial step in understanding TF functioning in the SG and provide useful information for further studies of the regulatory roles of TFs in the SG.

## Conclusion

In this study, we provide the first report of the systematic identification of TFs in the SG of *B*. *mori*, and we describe their characteristics and expression profiles in different tissues, with a focus on the SG. These results provide a fundamental understanding of the function and regulation of TFs in the SG. At present, we are investigating the functions and interactions of some candidate TFs expressed in the SG via the transgenic overexpression and CRISPR/Cas9-mediated specific knockout of TFs in the SG. We believe that these ongoing efforts will provide more reliable evidence to thoroughly answer the question of how the SG efficiently synthesizes silk proteins.

## Supporting information

S1 File Manually modified GFF file based on the complete silkworm genome sequence in KAIKObase. (GZ)

S2 File Distribution of conserved motifs predicted in TF DBD families. (PPTX)

S3 File Phylogenetic tree of nine TF DBD families. (PPTX)

S1 Table Primer sequences used for qRT-PCR detection. (DOCX)

S2 Table List of 348 TFs identified in the SG. (XLSX)

S3 Table List of 228 TFs expressed in the SG. (XLSX)
