# Supplementary material for: Genome-wide survey and characterization of transcription factors in the silk gland of the silkworm, Bombyx mori
Source: PLoS One. 2021 Nov 11;16(11):e0259870. doi: 10.1371/journal.pone.0259870 (PMC8584736; doi:10.1371/journal.pone.0259870)
Supplement: S2 File — (PPTX) [file pone.0259870.s002.pptx]

## Slide 1
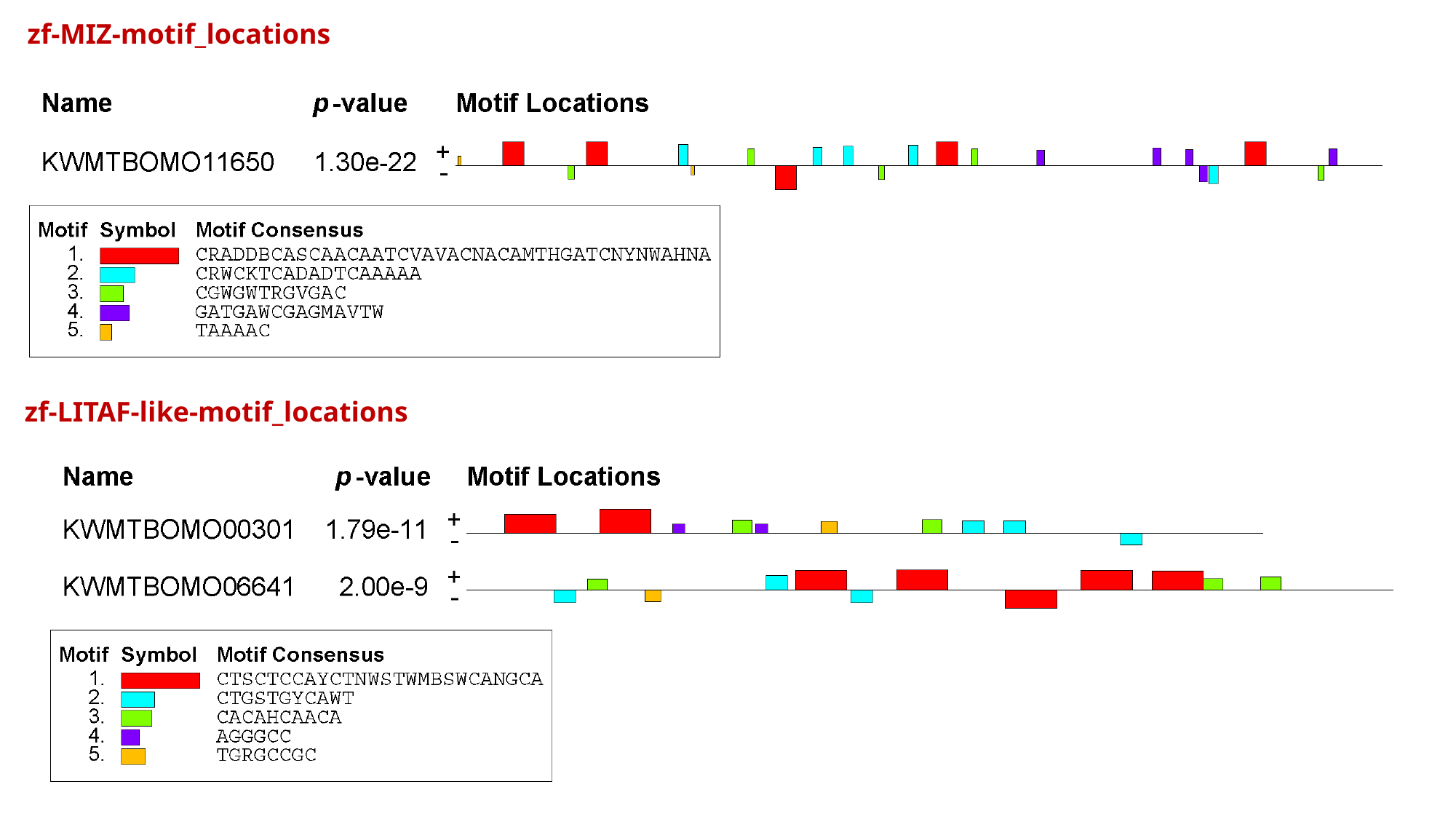

zf-MIZ-motif_locations
zf-LITAF-like-motif_locations

## Slide 2
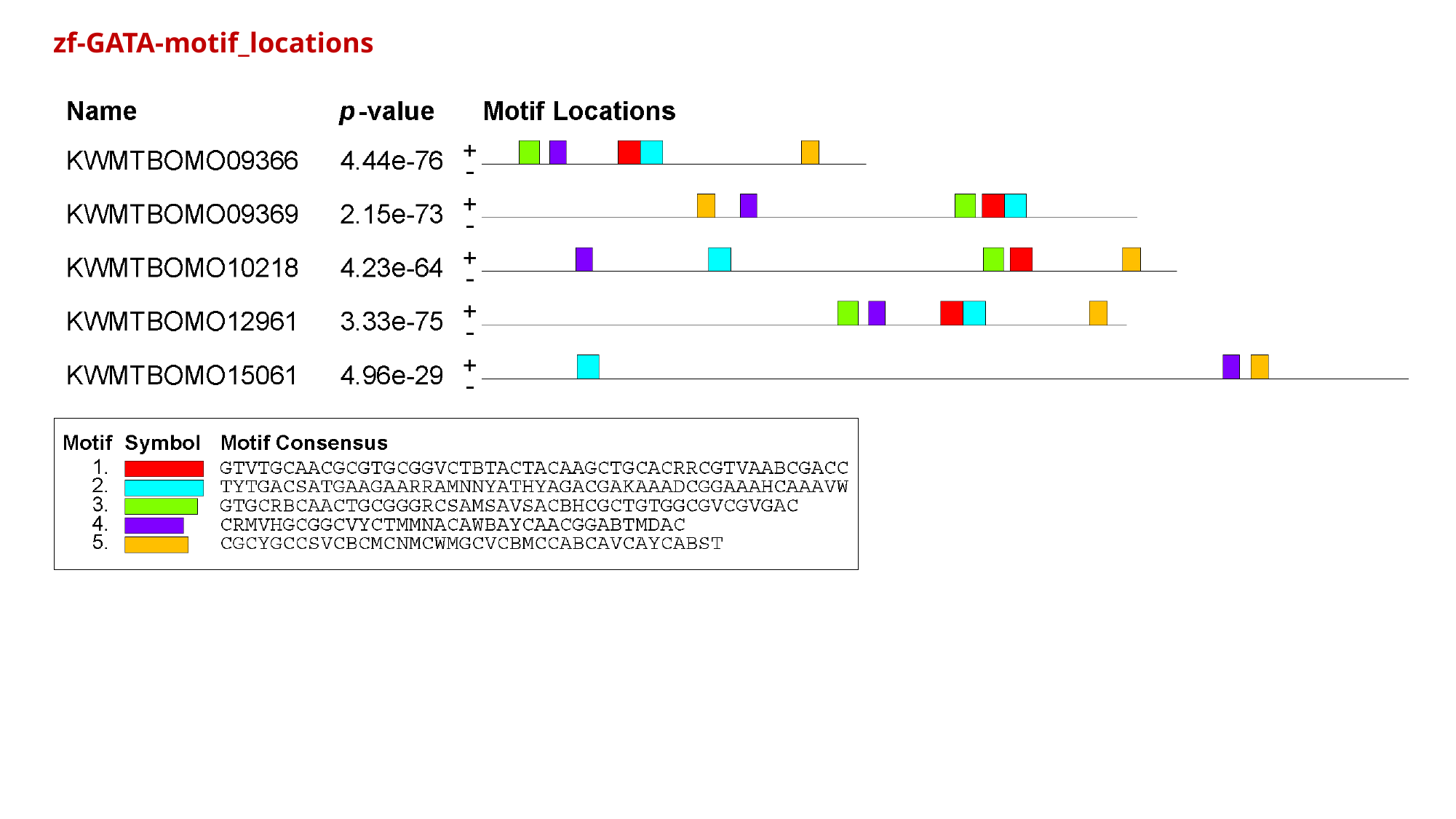

zf-GATA-motif_locations

## Slide 3
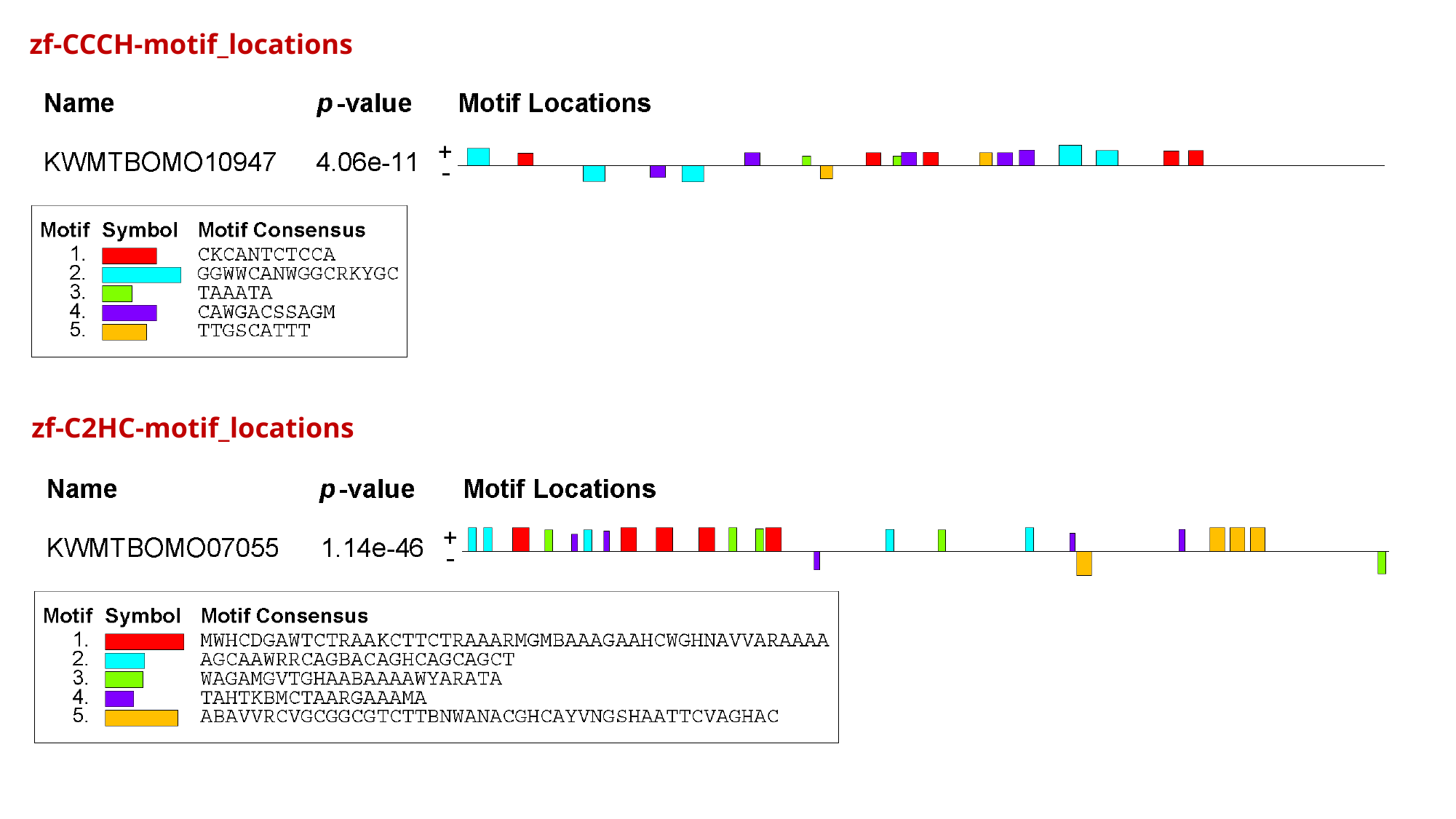

zf-CCCH-motif_locations
zf-C2HC-motif_locations

## Slide 4
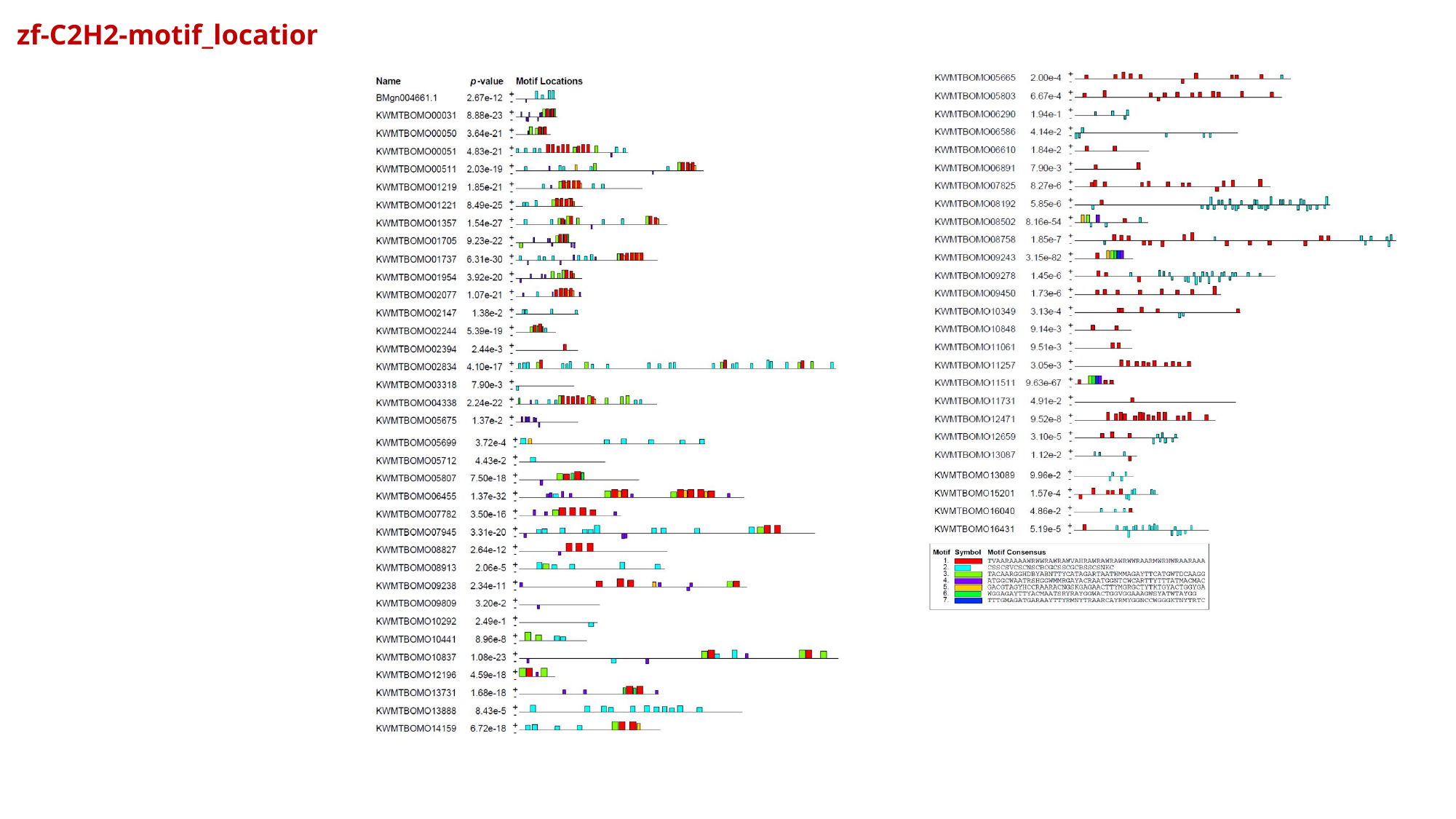

zf-C2H2-motif_locations

## Slide 5
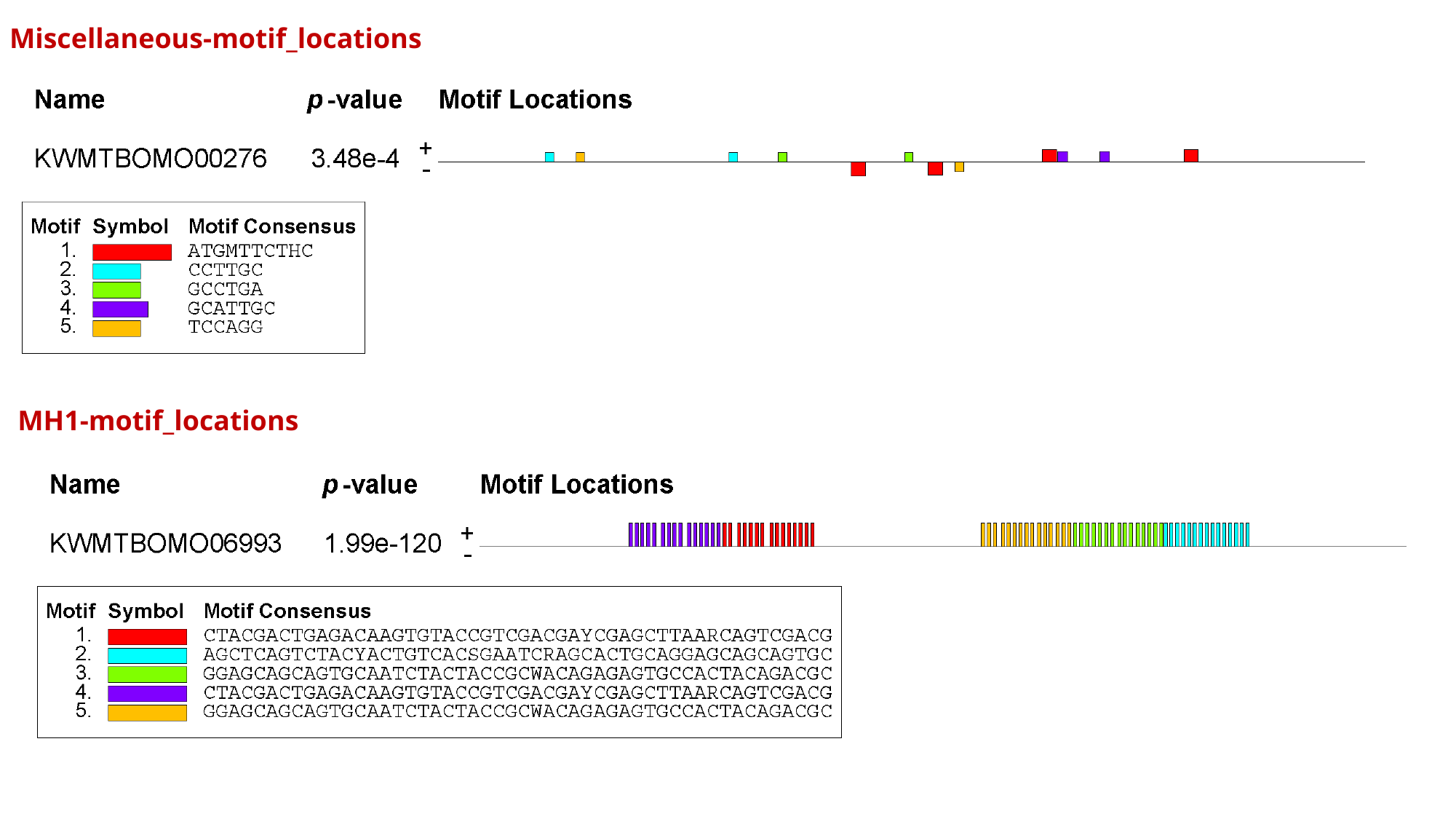

Miscellaneous-motif_locations
MH1-motif_locations

## Slide 6
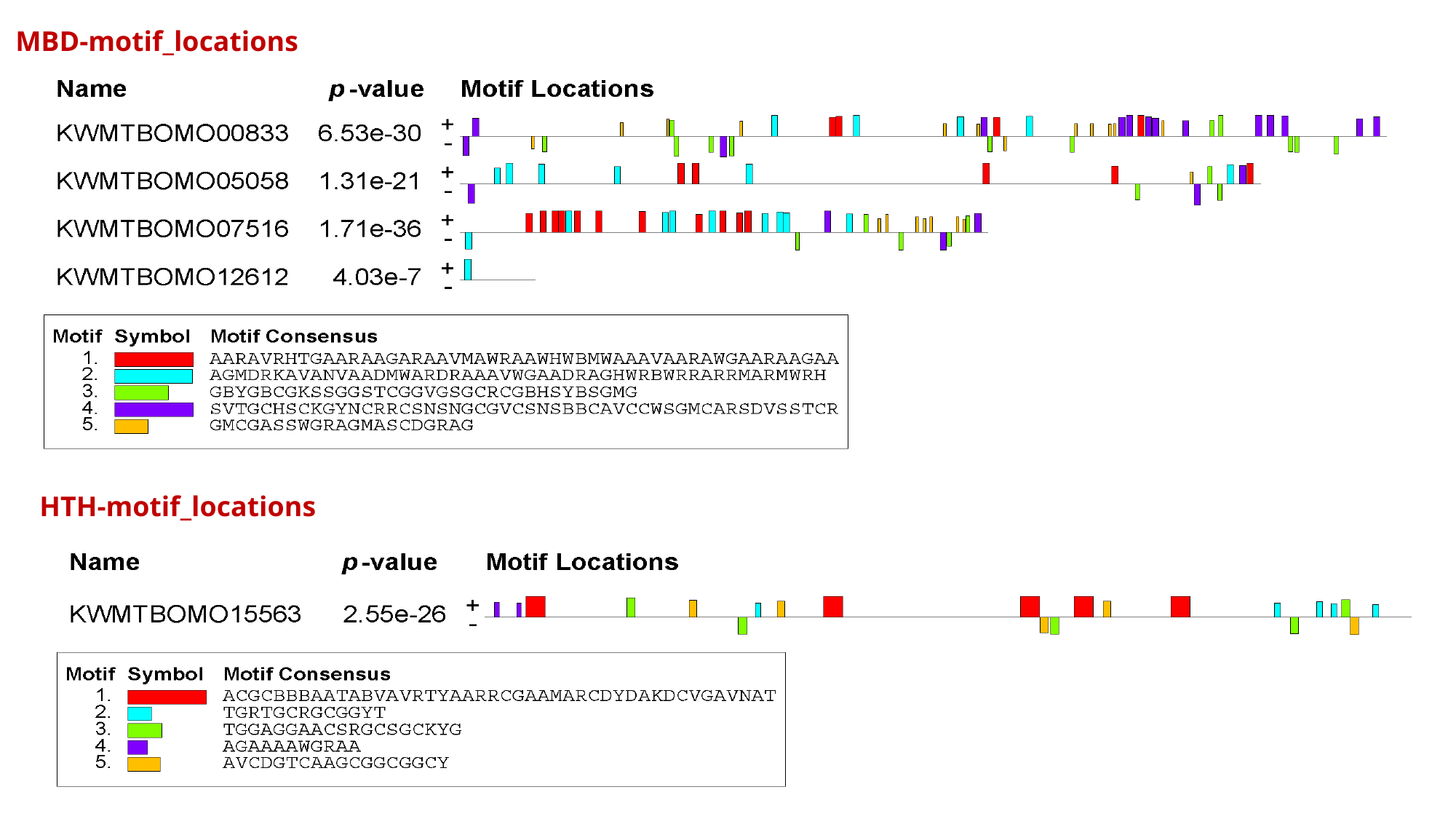

MBD-motif_locations
HTH-motif_locations

## Slide 7
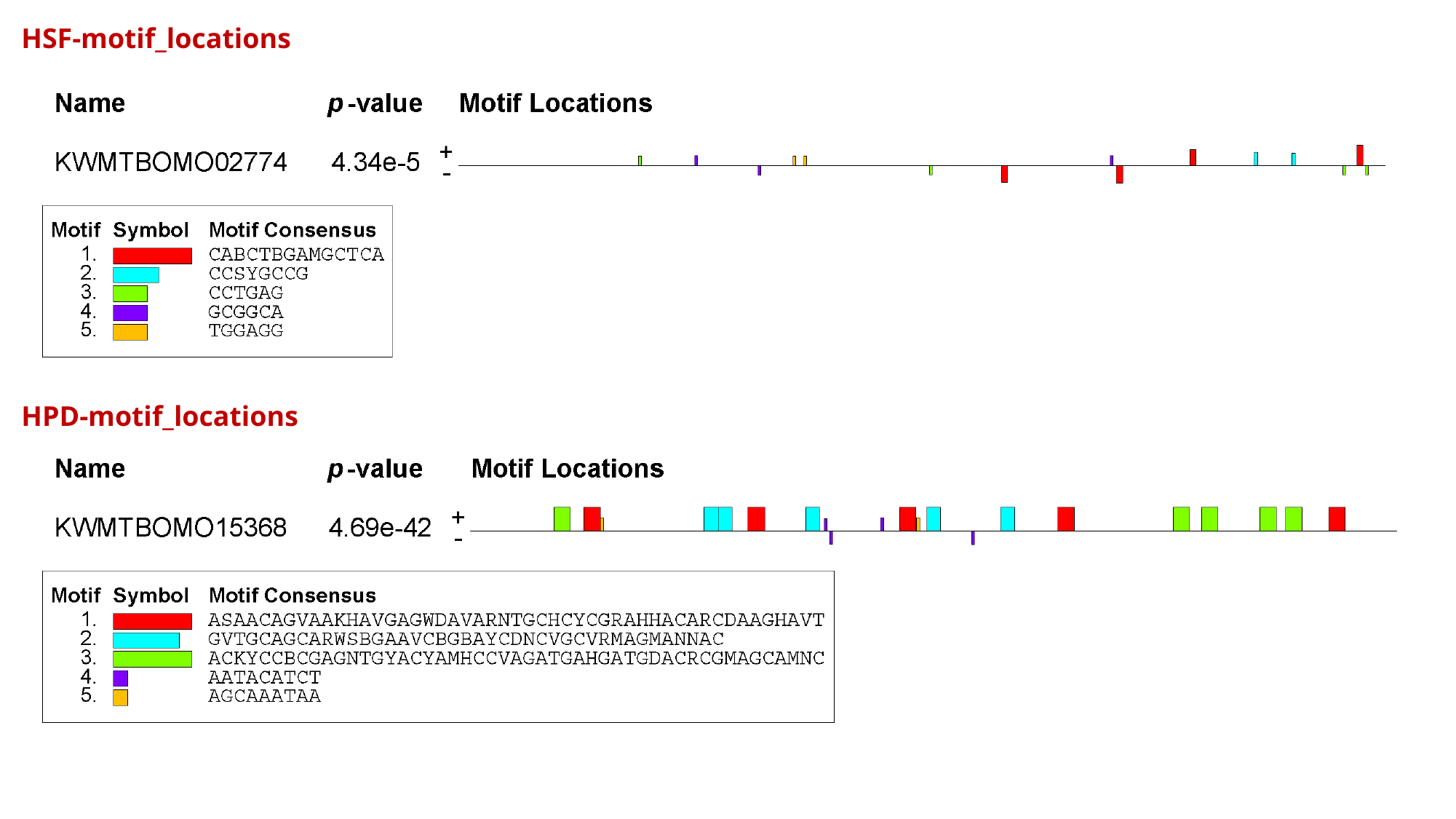

HSF-motif_locations
HPD-motif_locations

## Slide 8
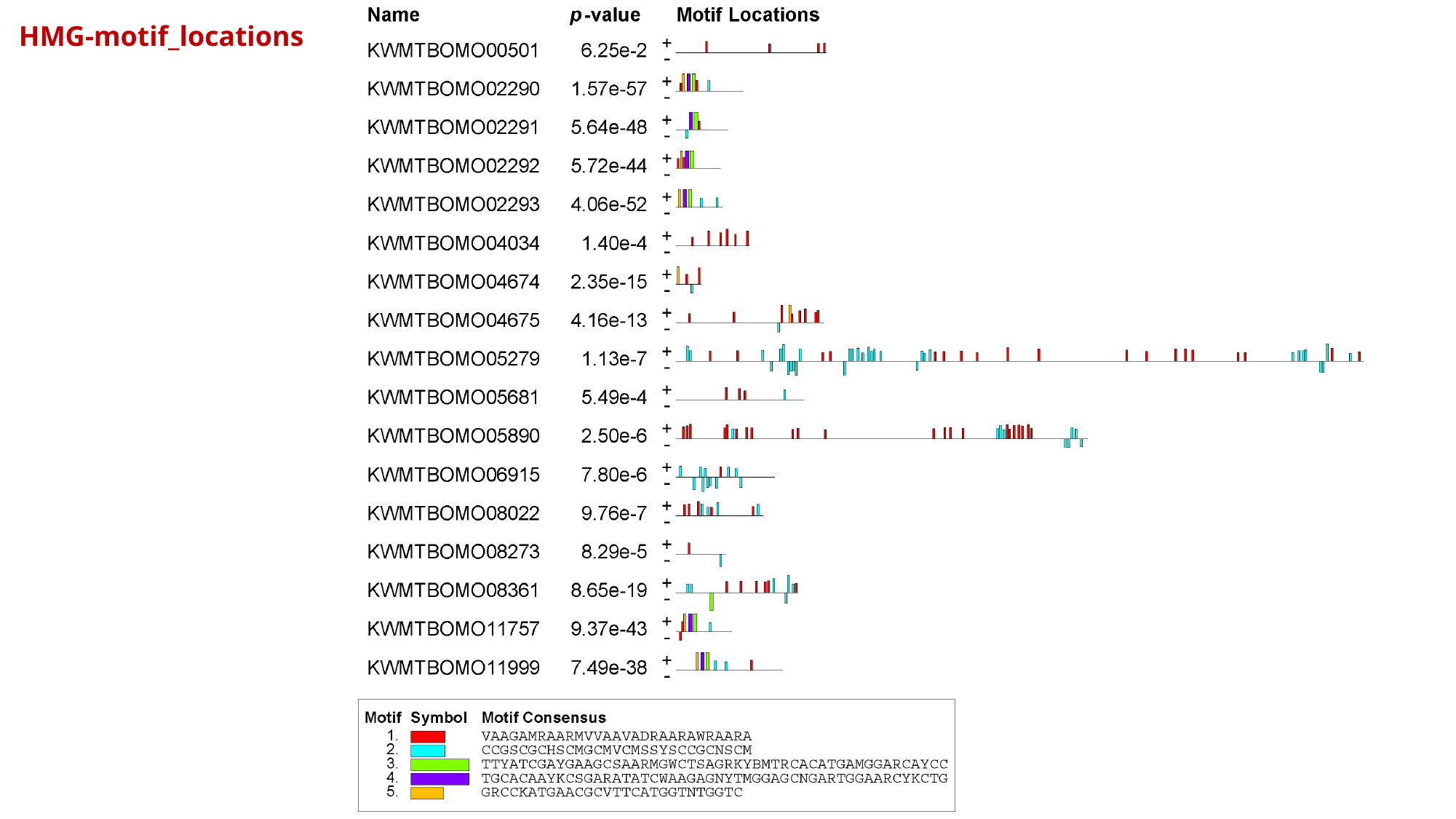

HMG-motif_locations

## Slide 9
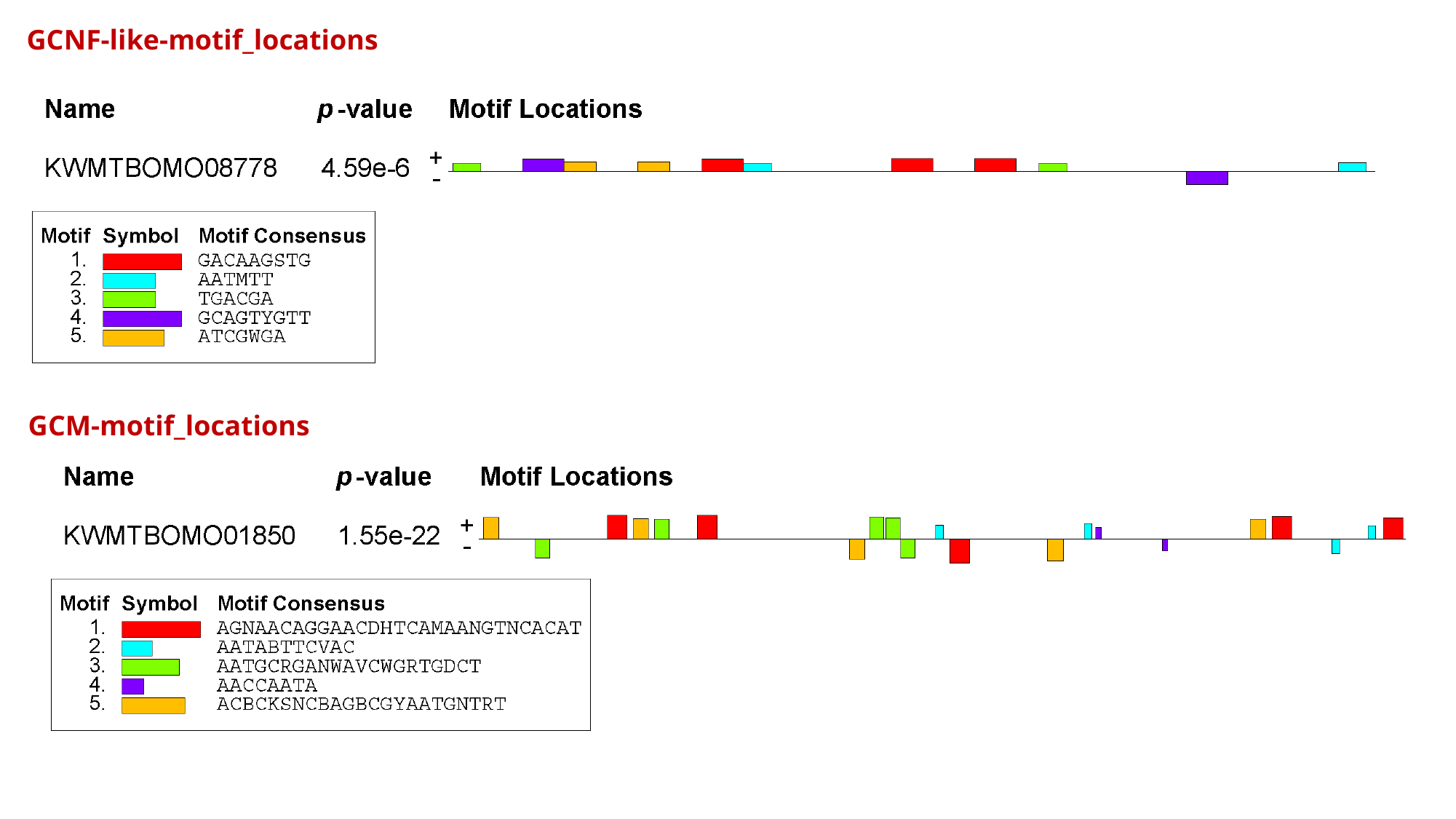

GCNF-like-motif_locations
GCM-motif_locations

## Slide 10
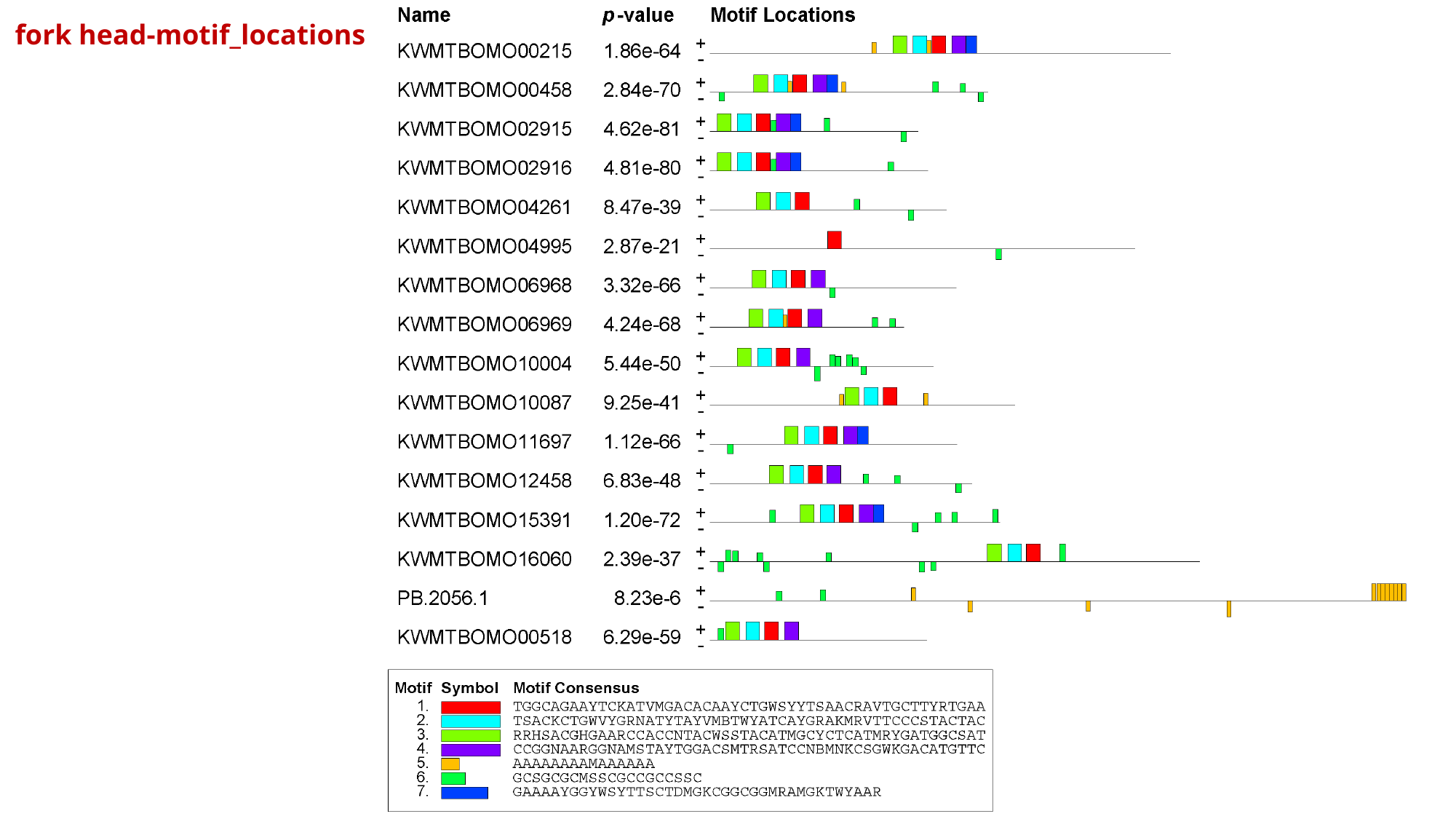

fork head-motif_locations

## Slide 11
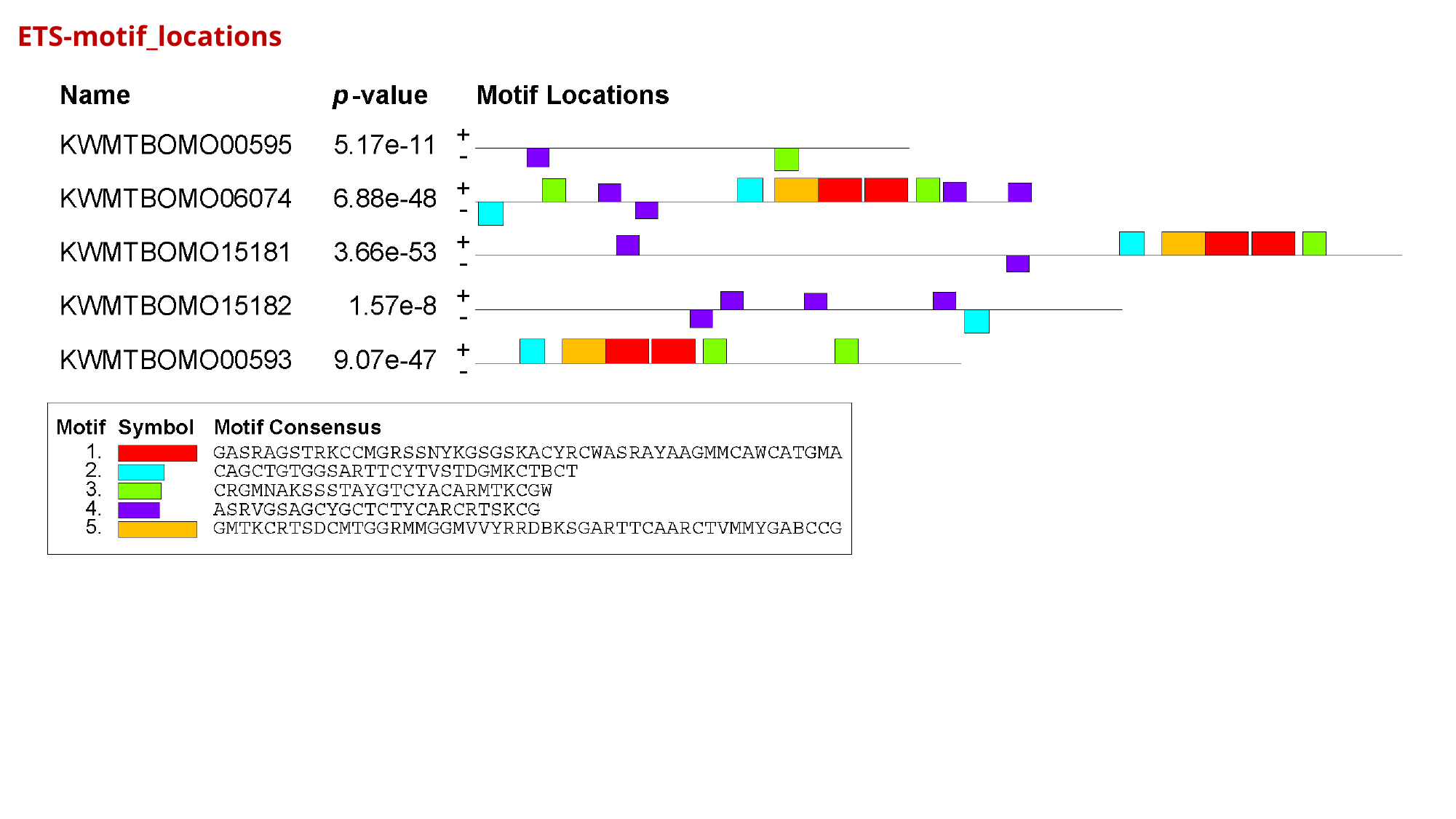

ETS-motif_locations

## Slide 12
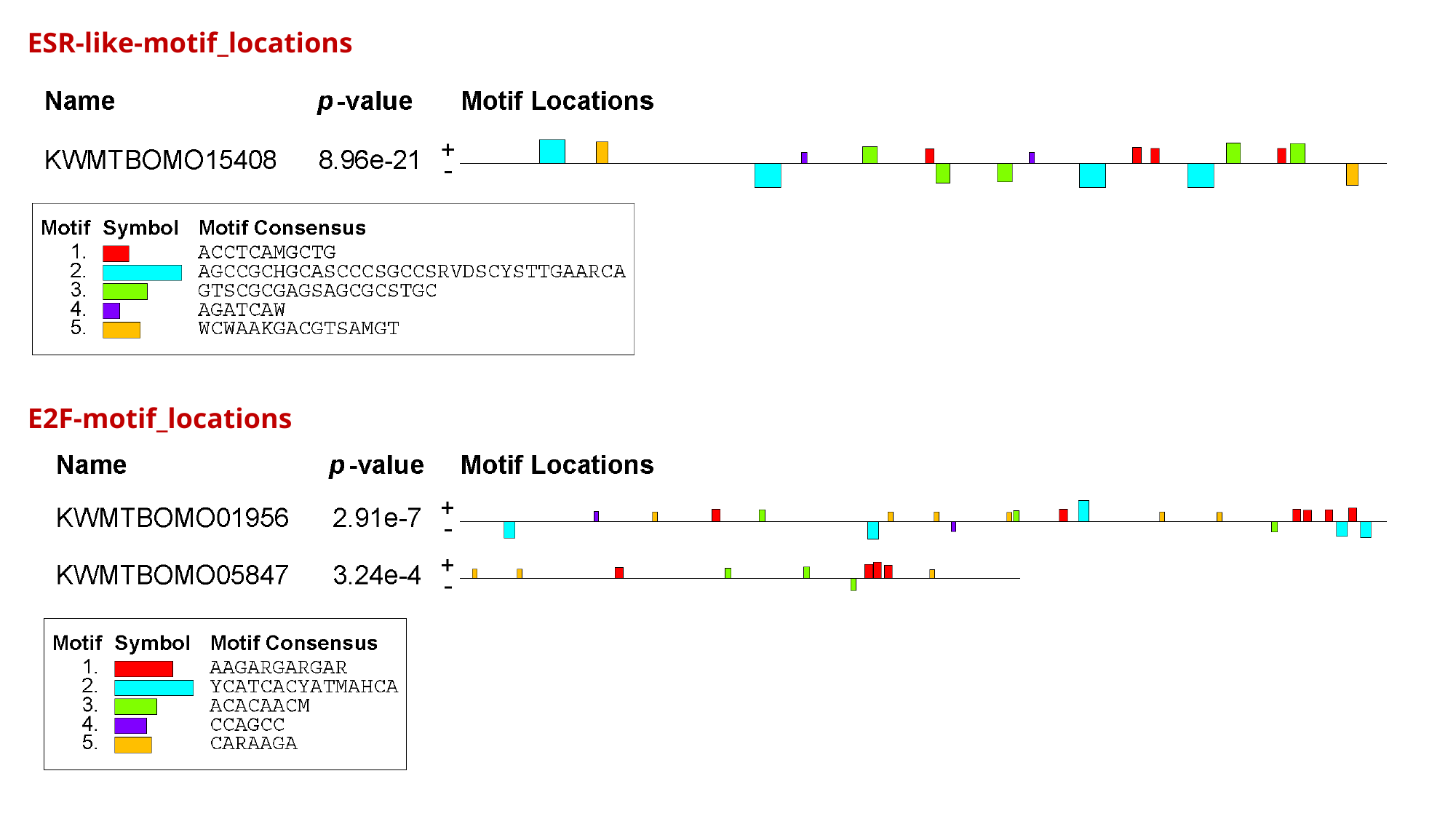

ESR-like-motif_locations
E2F-motif_locations

## Slide 13
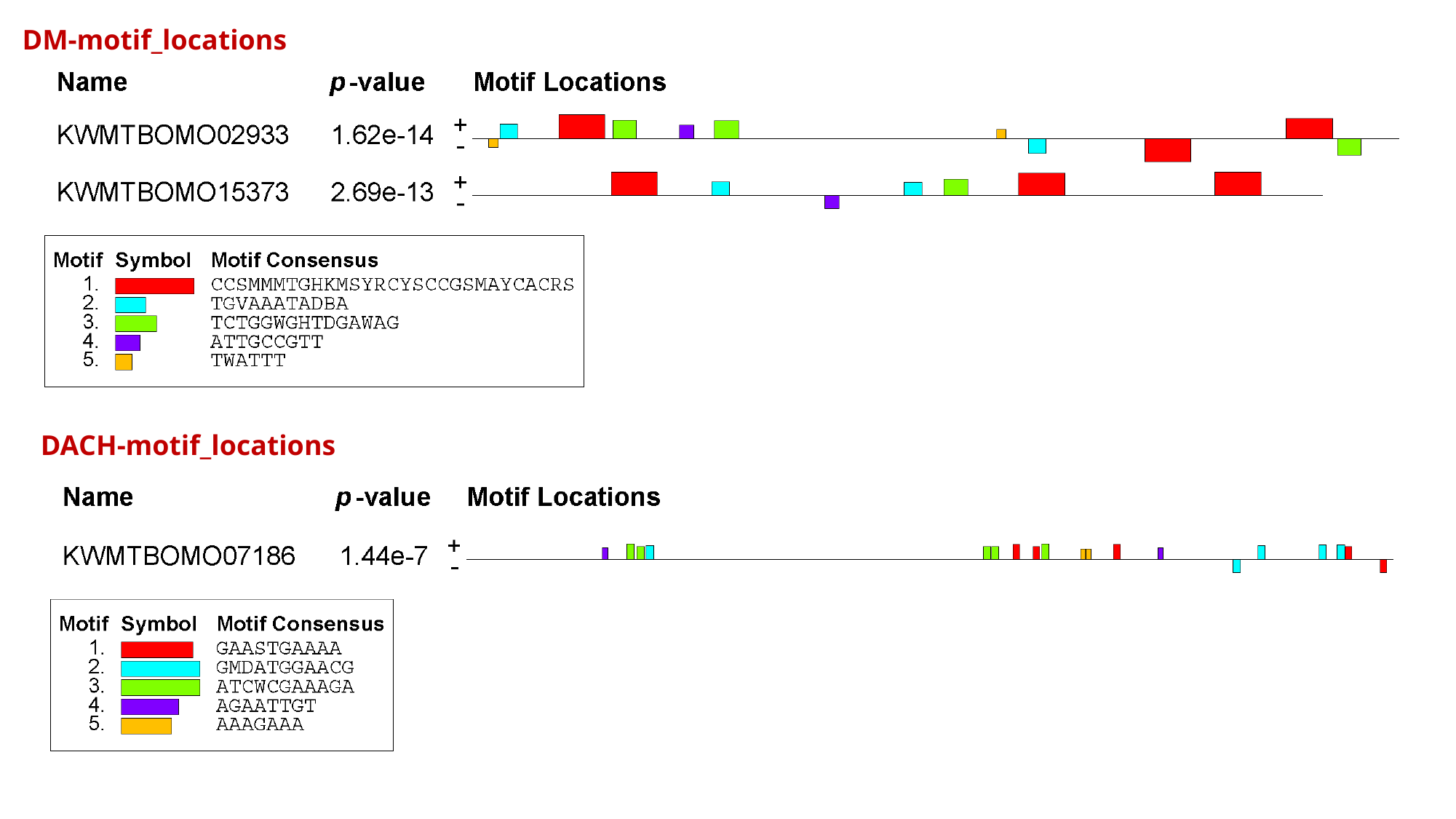

DM-motif_locations
DACH-motif_locations

## Slide 14
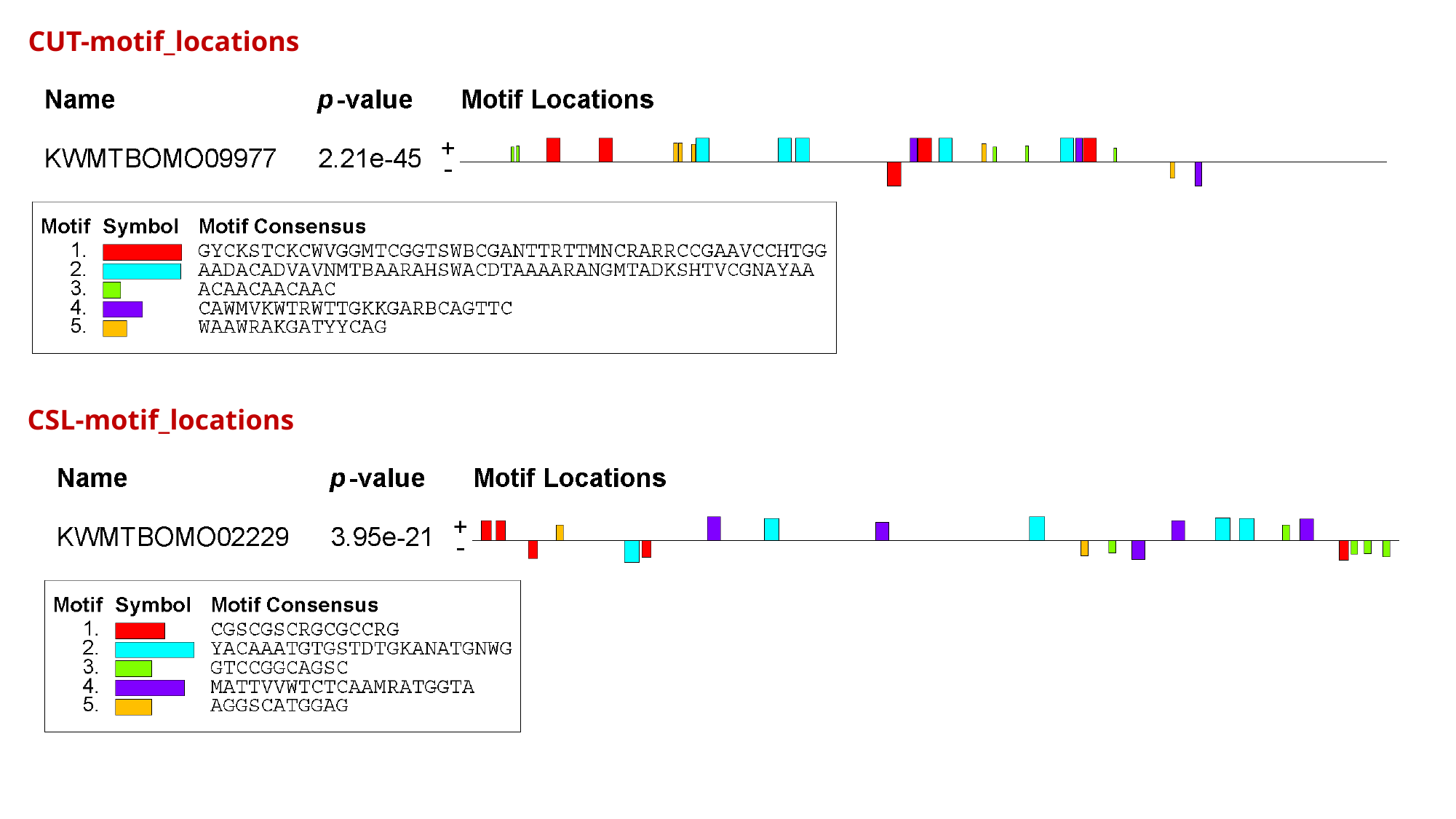

CUT-motif_locations
CSL-motif_locations

## Slide 15
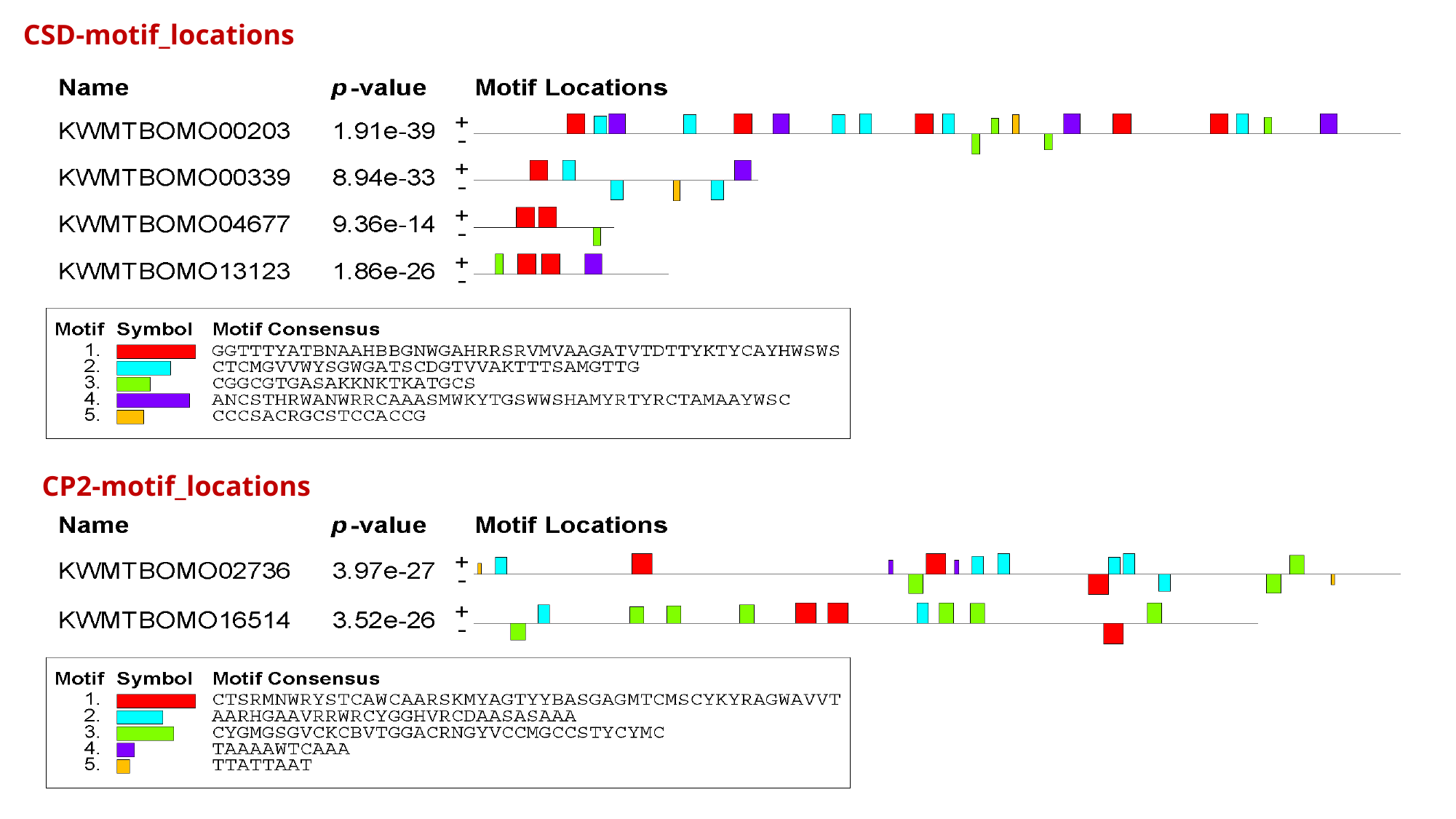

CSD-motif_locations
CP2-motif_locations

## Slide 16
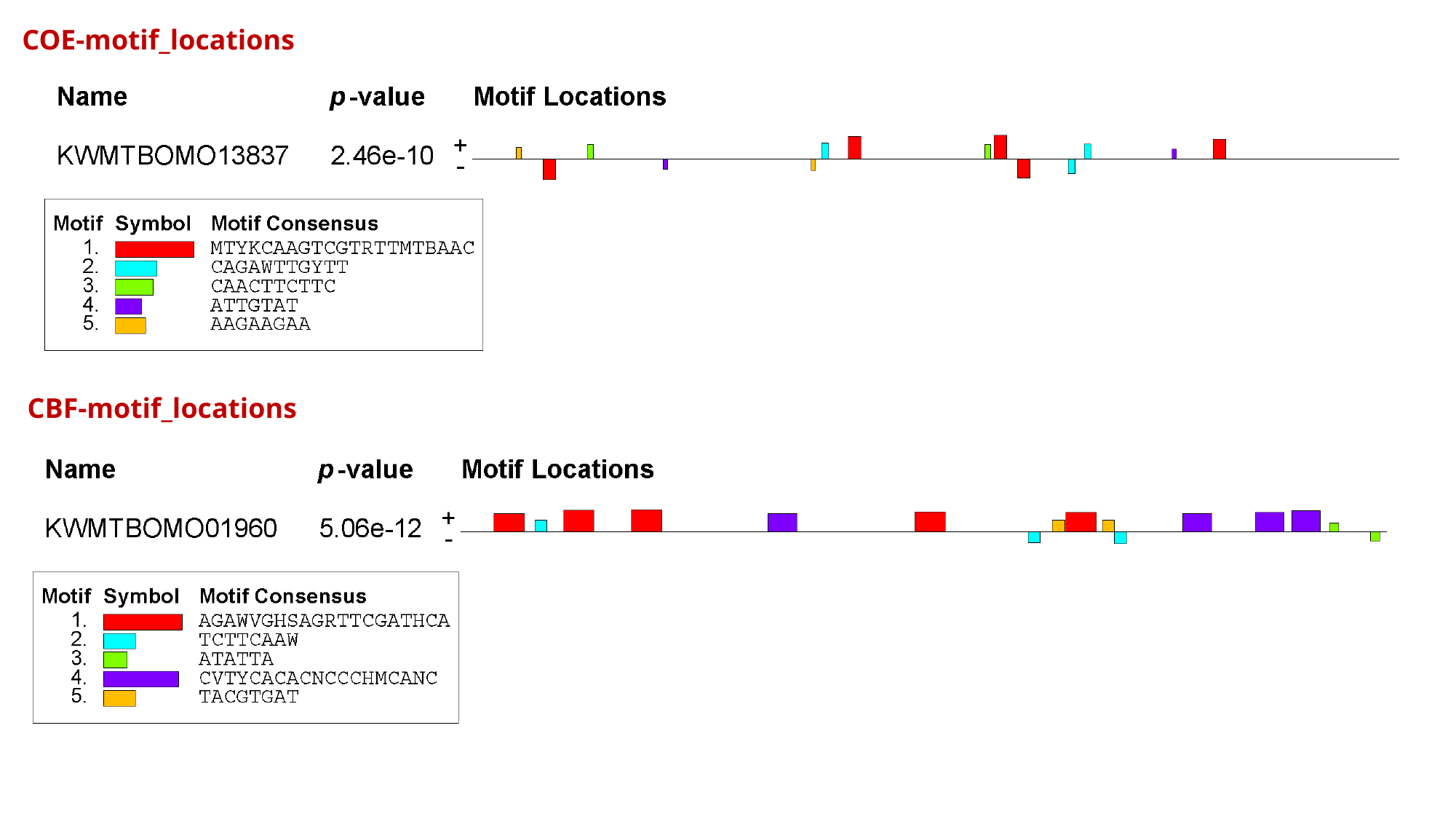

COE-motif_locations
CBF-motif_locations

## Slide 17
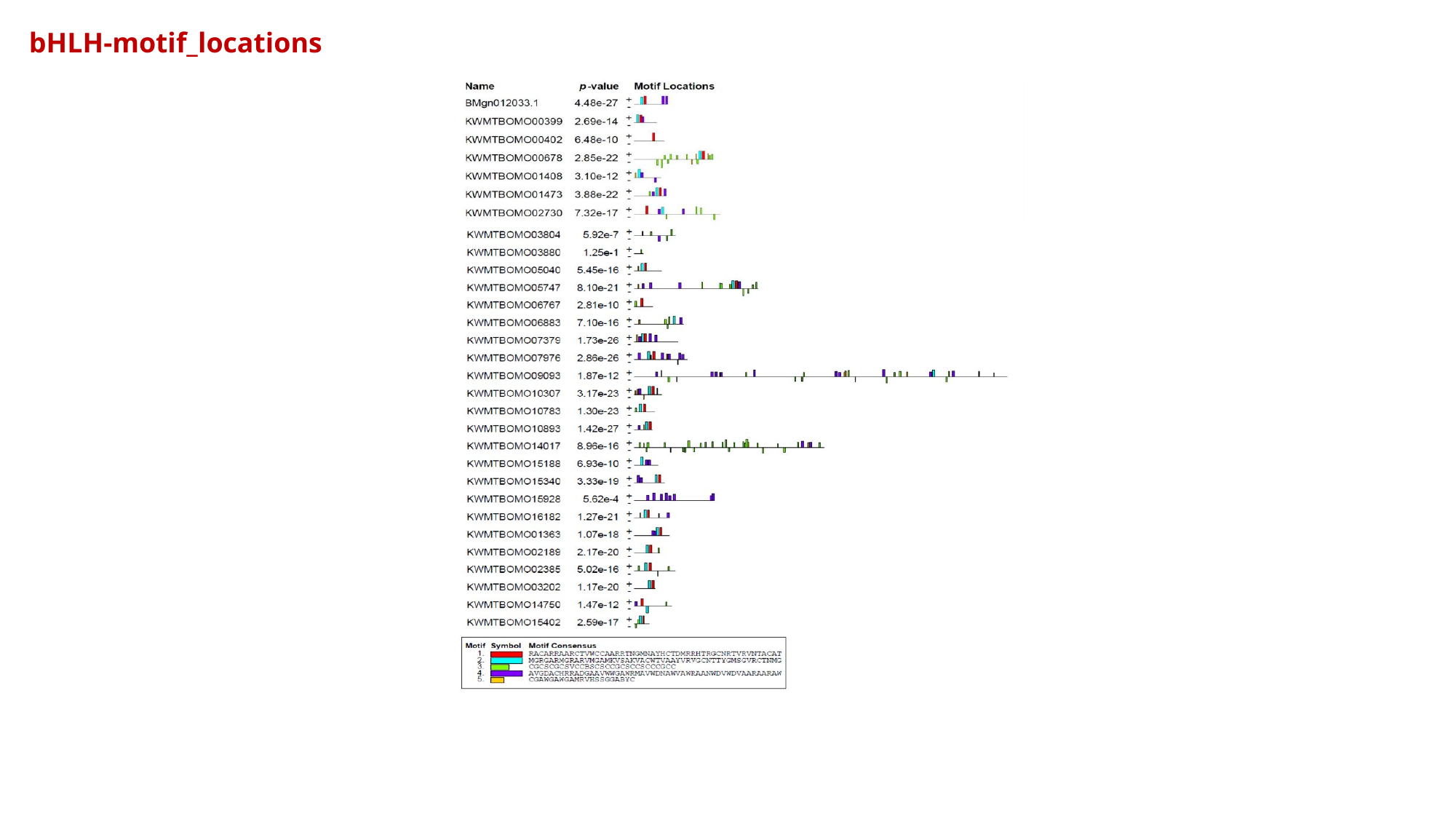

bHLH-motif_locations

## Slide 18
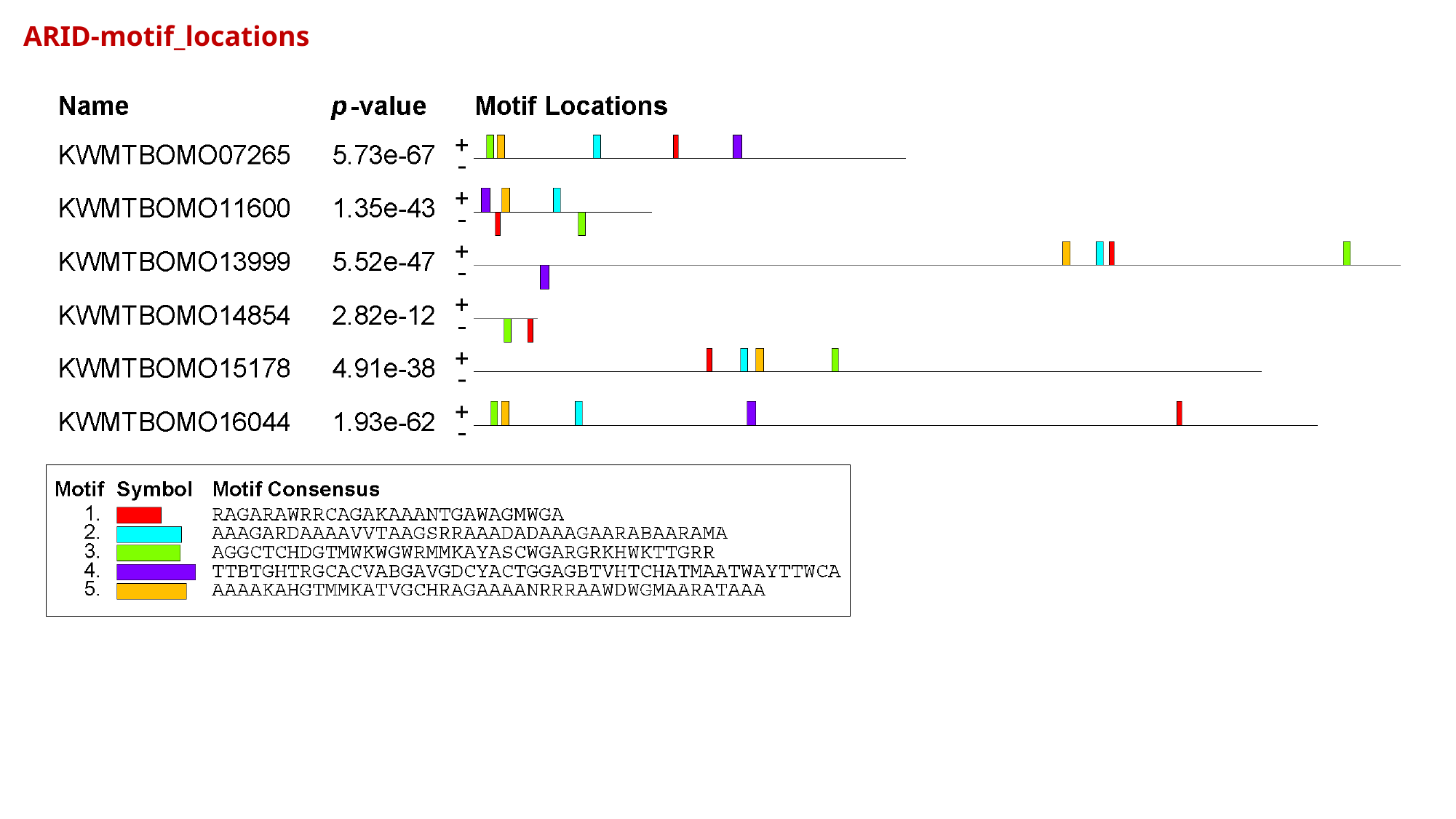

ARID-motif_locations

## Slide 19
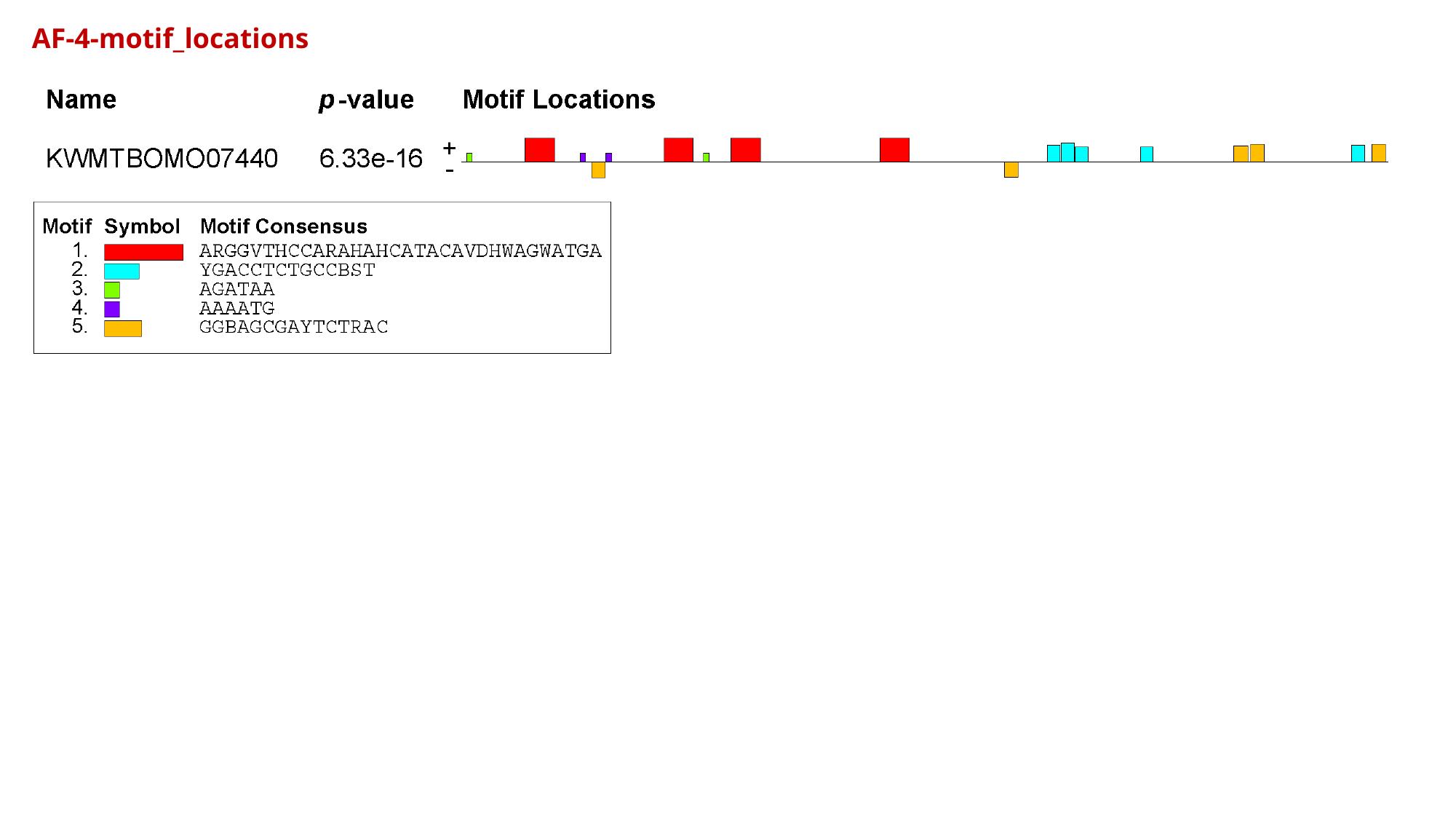

AF-4-motif_locations
