# Supplementary material for: Genome-wide survey and characterization of transcription factors in the silk gland of the silkworm, Bombyx mori
Source: PLoS One. 2021 Nov 11;16(11):e0259870. doi: 10.1371/journal.pone.0259870 (PMC8584736; doi:10.1371/journal.pone.0259870)
Supplement: S3 File — (PPTX) [file pone.0259870.s003.pptx]

## Slide 1
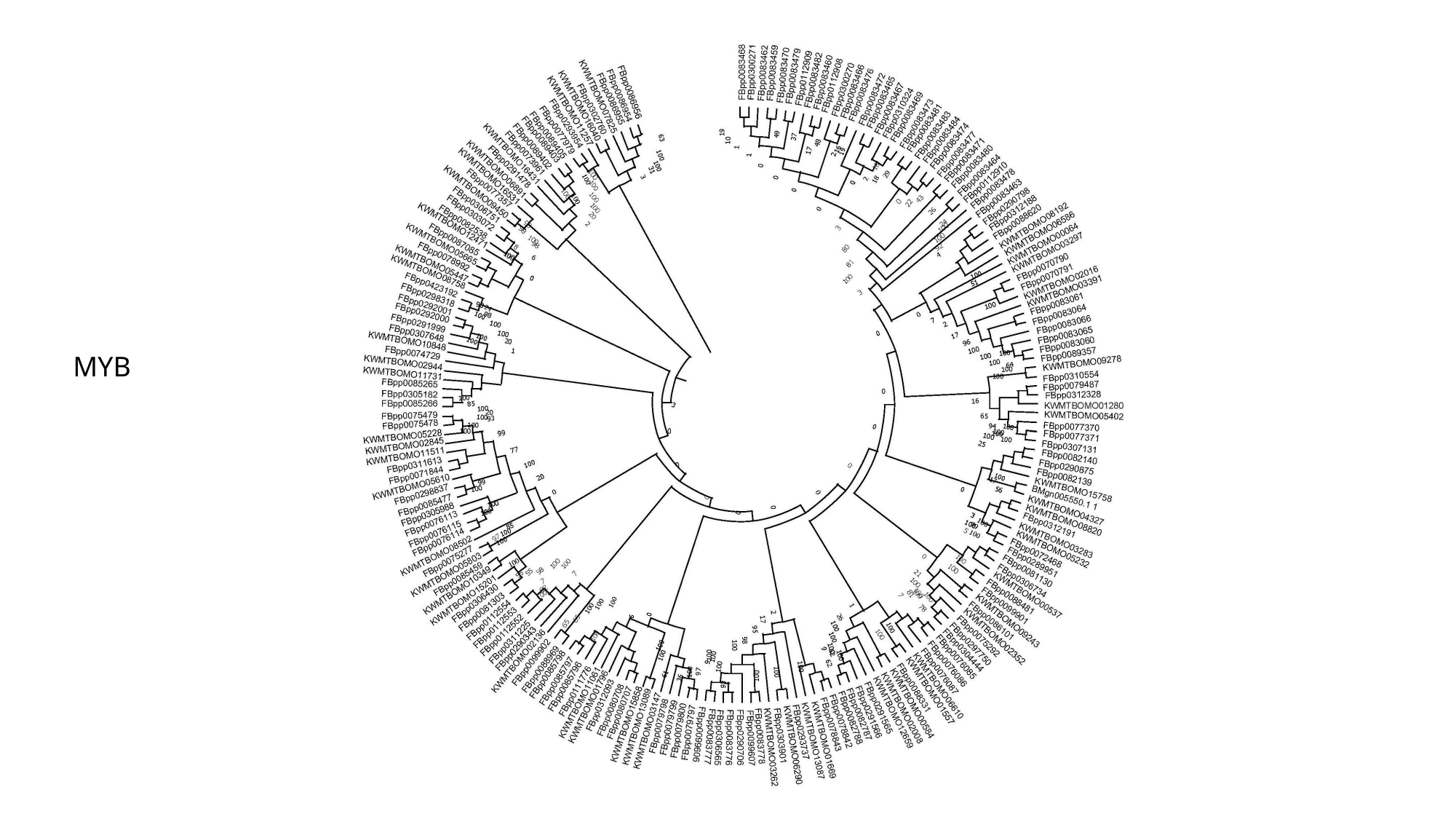

MYB

## Slide 2
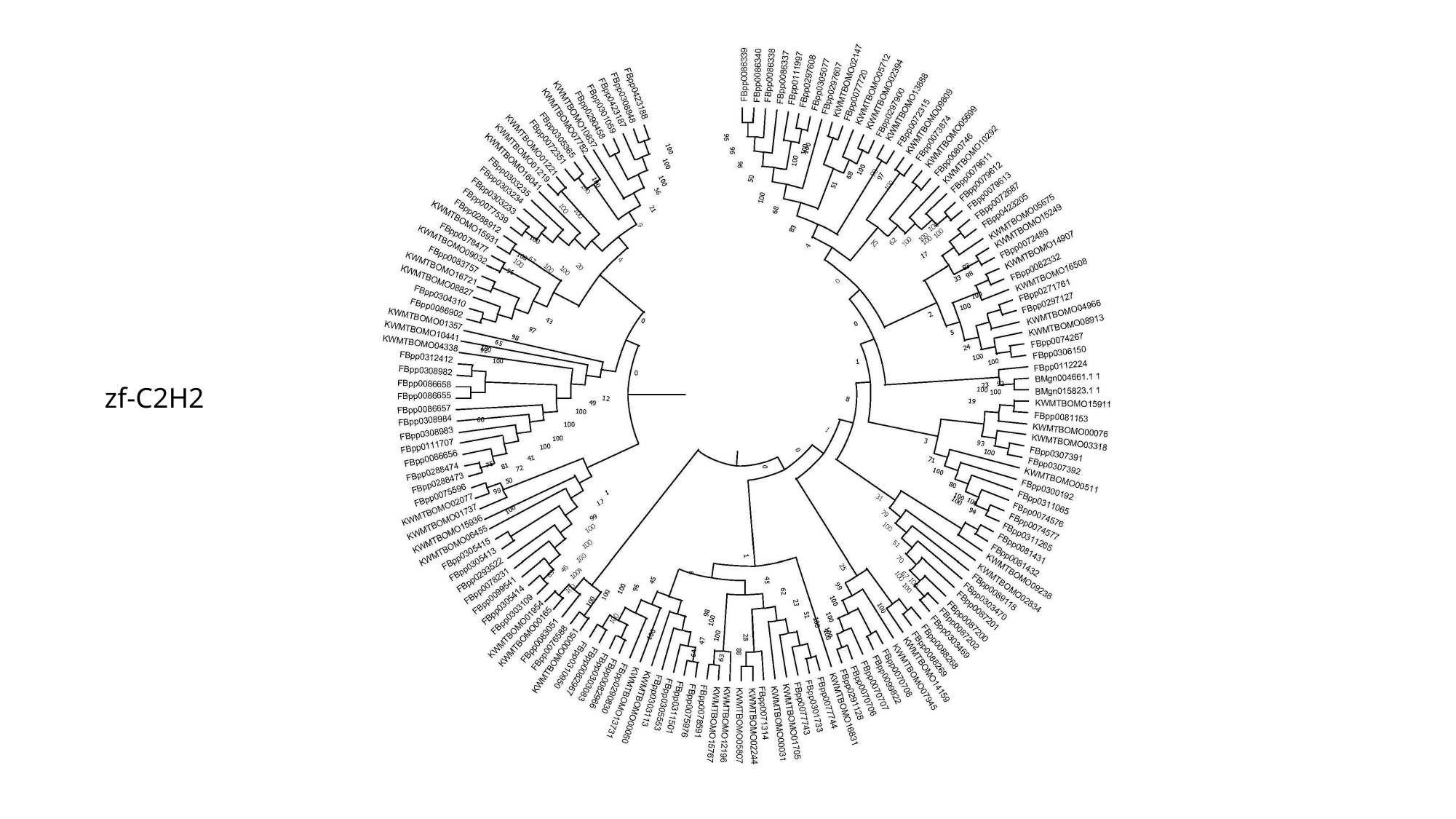

zf-C2H2

## Slide 3
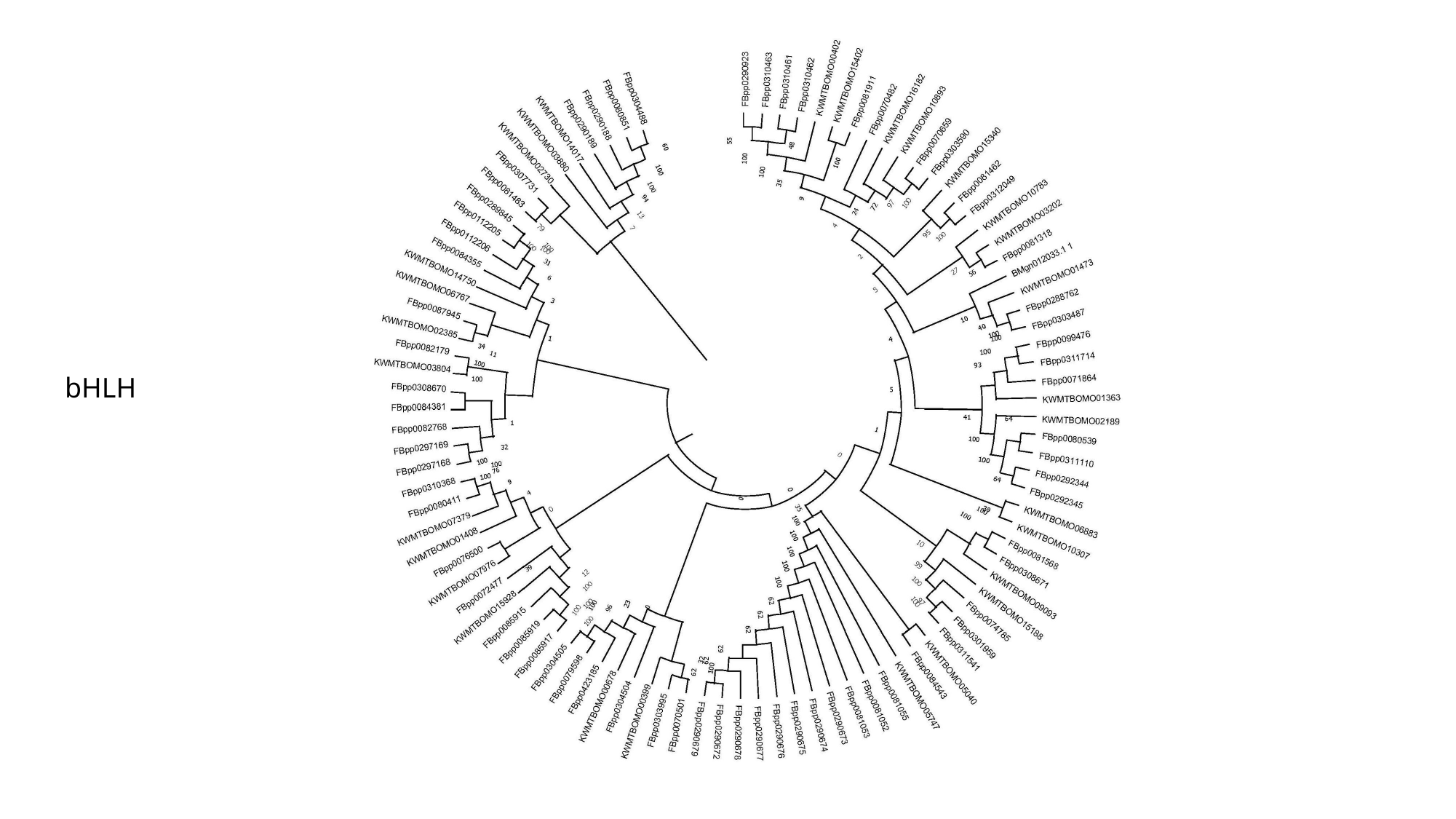

bHLH

## Slide 4
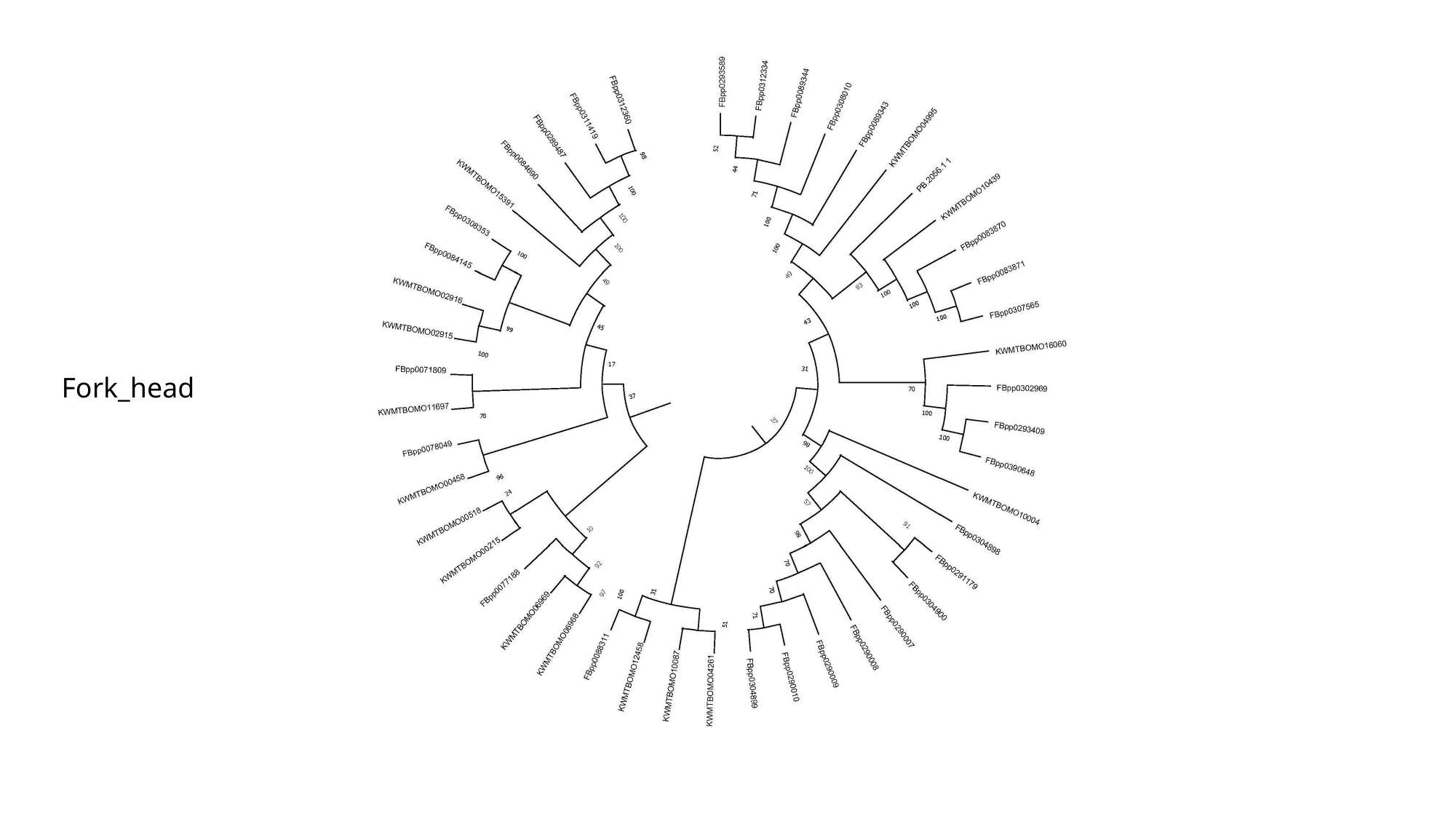

Fork_head

## Slide 5
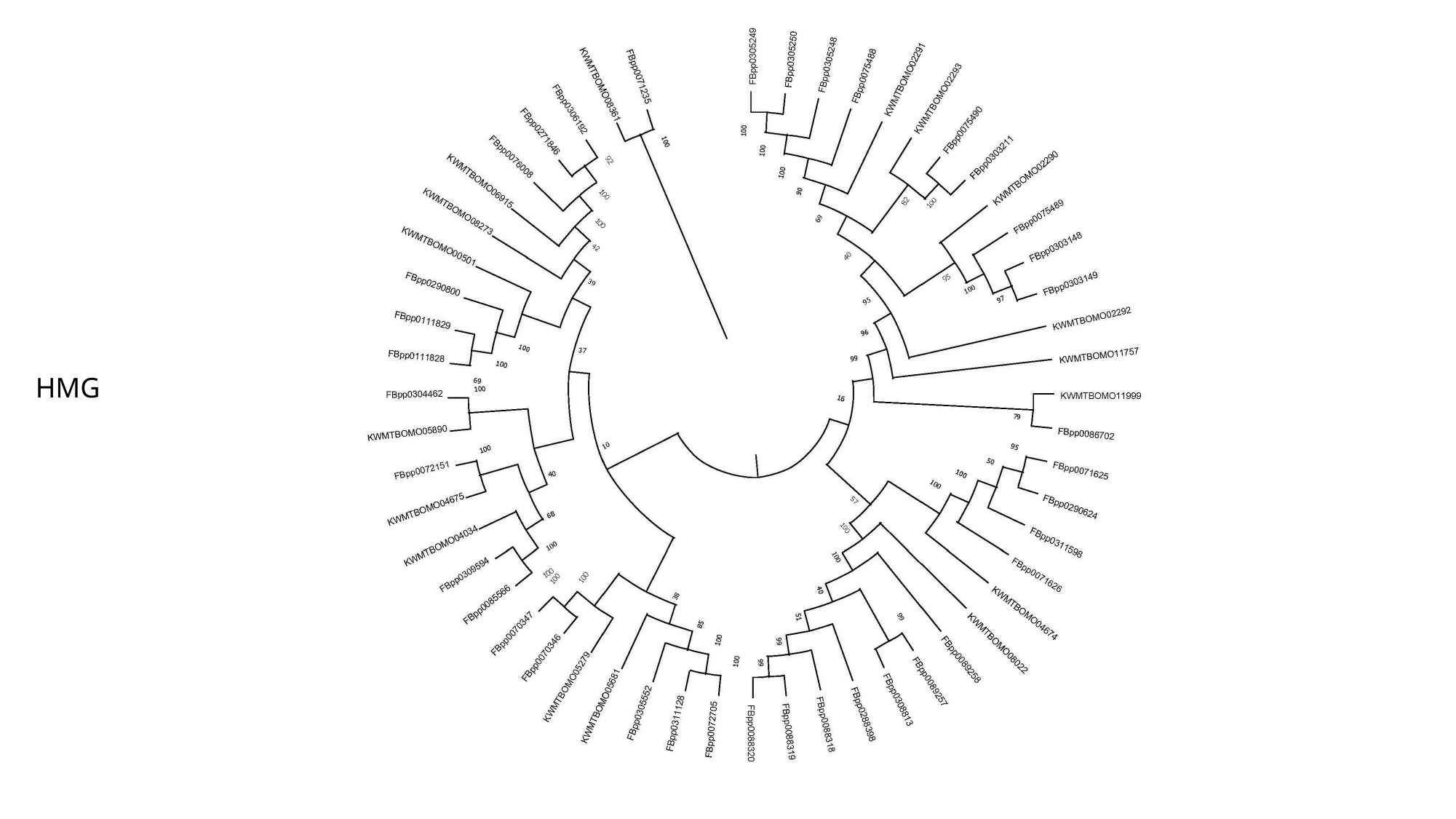

HMG

## Slide 6
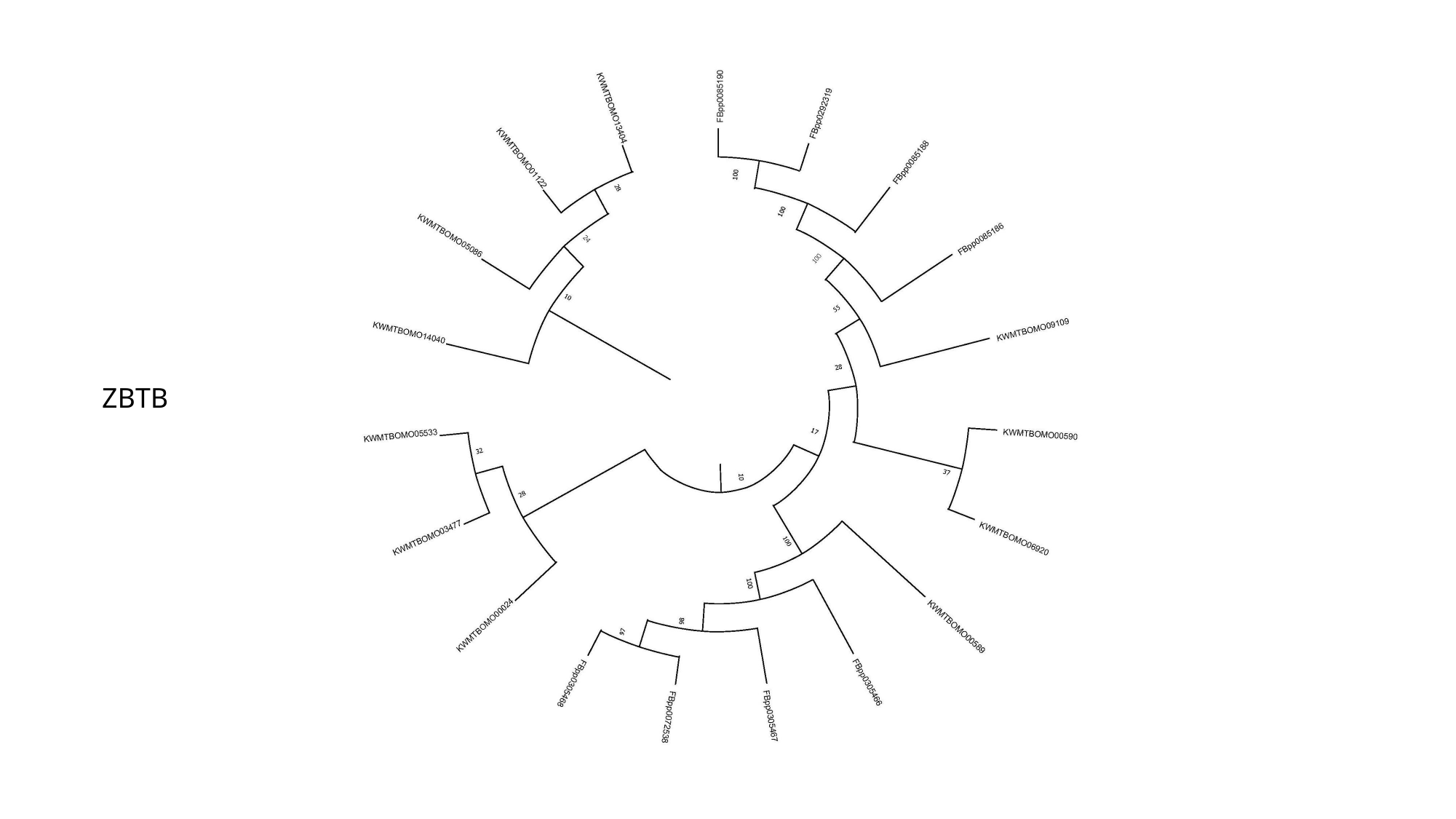

ZBTB

## Slide 7
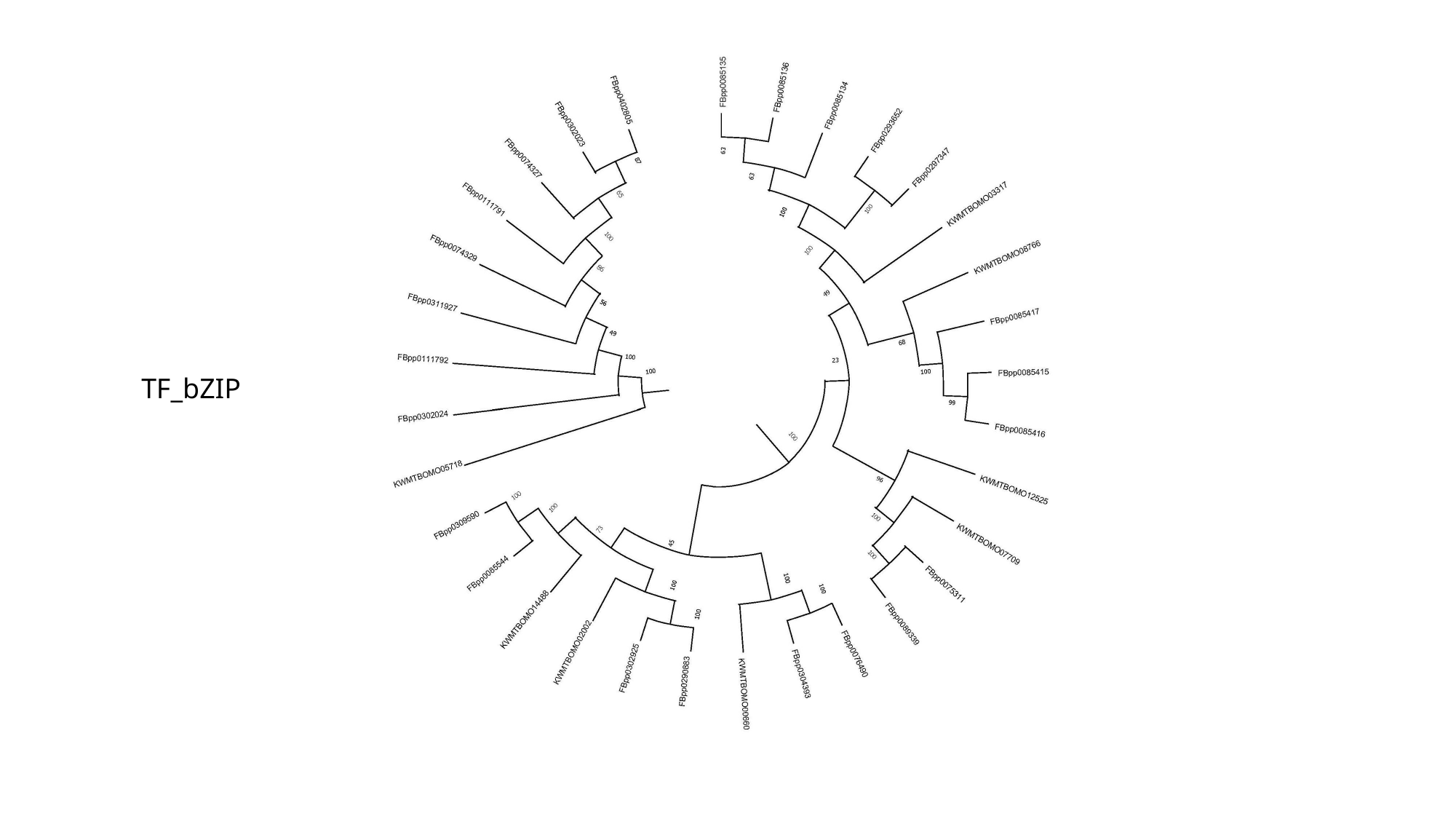

TF_bZIP

## Slide 8
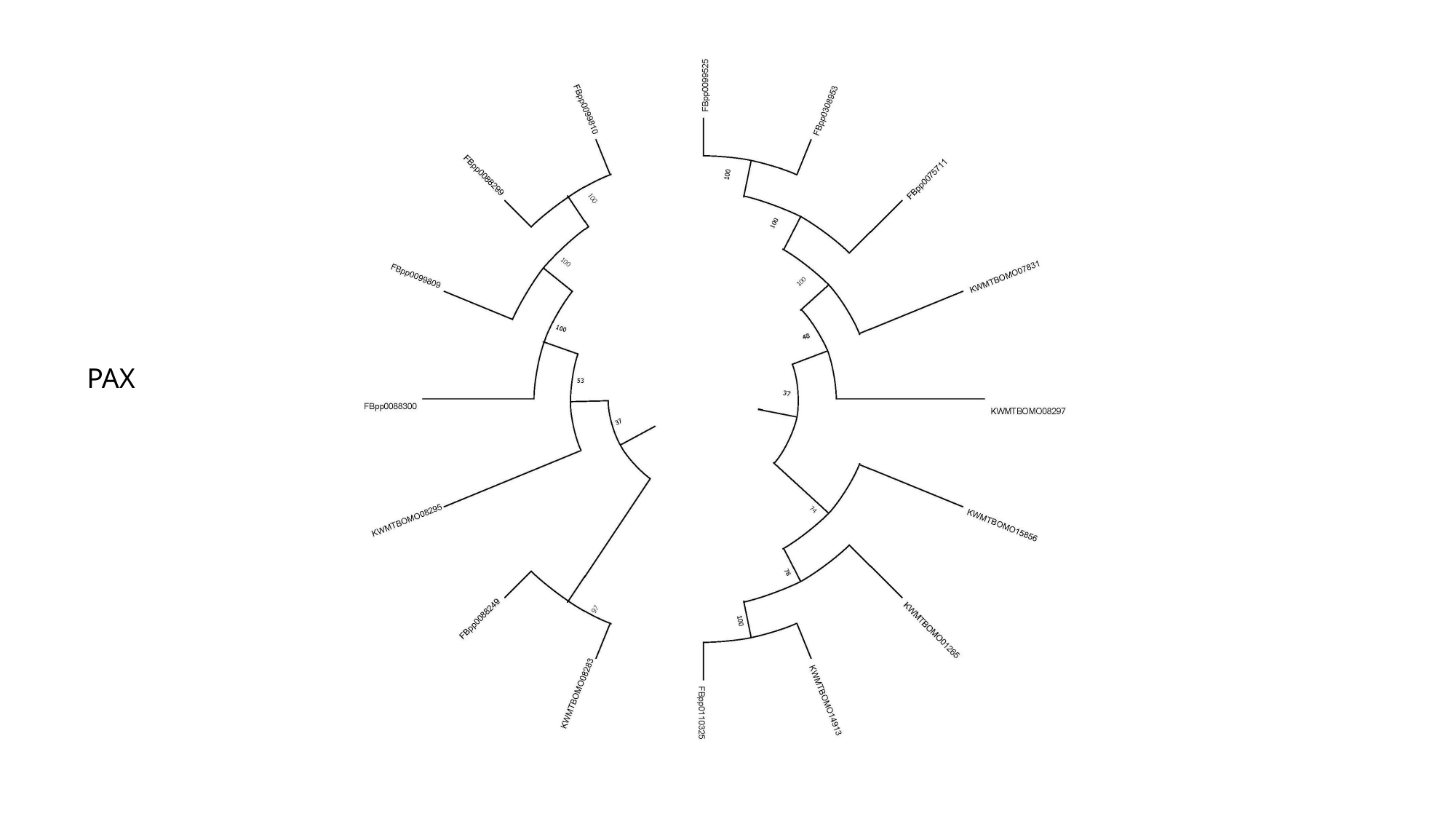

PAX

## Slide 9
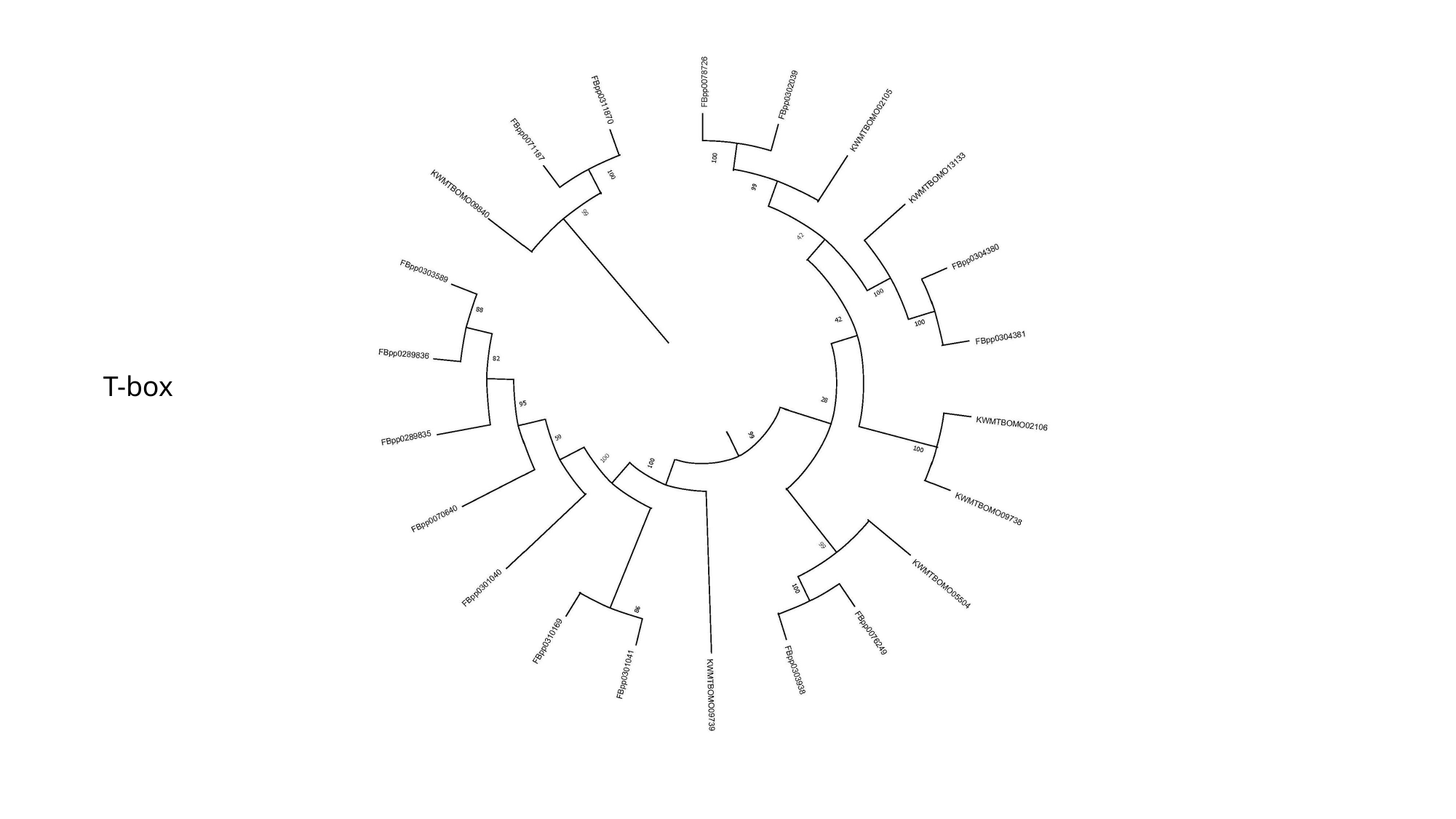

T-box
