# Supplementary material for: Genome-wide survey and characterization of transcription factors in the silk gland of the silkworm, Bombyx mori
Source: PLoS One. 2021 Nov 11;16(11):e0259870. doi: 10.1371/journal.pone.0259870 (PMC8584736; doi:10.1371/journal.pone.0259870)
Supplement: S1 Table — (DOCX) [file pone.0259870.s004.docx]

**S1 Table. Primer sequences used for qRT-PCR detection**

| **Gene ID in silkbase** | **Name** | **Primer sequences (5’ - 3’)** |
| --- | --- | --- |
| KWMTBOMO15340 | Sage | F: GAGCCTAGCAATCACGAAGG  R: TTCCAACTGCAAGGCTCTTT |
| KWMTBOMO08651 | Exd | F: GCACCAGACCTATCACCCCT  R: CGCTTGCTTGCTGAAGTTAC |
| KWMTBOMO05671 | E75A | F: CTACGTCCGTCGACCACTCT  R: CAGGGCCGGTATTGTATCTT |
| KWMTBOMO15391 | SGF1 | F: GCAGCACCCGTTCAGCATC  R: GCGGCGACTGGTAGTAGTTATCC |
| KWMTBOMO13459 | SGF3 | F: GAAACCGTCCGCTCAAGAAA  R: GTTCGGTGGCGTCATCCTC |
| KWMTBOMO08597 | E74B | F: TACCTGTGGGAGTTCCTGCT  R: TCCCGCGCTGGTAGTAATAG |
| KWMTBOMO15563 | E93 | F: CCCCAAATTCTTGGATAGCA  R: GCCGAACTTGTTGACCTTGT |
| KWMTBOMO08031 | Sd | F: AGTCCCGACATCGAACAGAG  R: TTCGCCTAGCTAGCACCTGT |
| KWMTBOMO00651 | Awh | F: AGAGTTTCGGTGCGAAGTGT  R: CGTCCAAGGTTTCGAGGTAG |
| KWMTBOMO05605 | EcRA | F: ATGGAGCTGAAACACGAGGT  R: TGAGGATAGTGGCGACGAGT |
| KWMTBOMO08726 | HR39 | F: TGTGGAACACTTGTGGCAAT  R: GCGGTAAACTCTTGCACCAT |
| KWMTBOMO00829 | HR3 | F: CAGGGTTCTTGGACGCAGACTTC  R: AGTCCAACCACATCTCCTCGTAG |
|  | BmeIF4A | F：TTCGTACTGGCTCTTCTCGT  R：CAAAGTTGATAGCAATTCCCT |
